# A geminivirus betasatellite encoded βC1 protein interacts with PsbP and subverts PsbP‐mediated antiviral defence in plants

**DOI:** 10.1111/mpp.12804

**Published:** 2019-04-15

**Authors:** Prabu Gnanasekaran, Kalaiarasan Ponnusamy, Supriya Chakraborty

**Affiliations:** ^1^ Molecular Virology Laboratory, School of Life Sciences Jawaharlal Nehru University New Delhi 110 067 India; ^2^ Synthetic Biology Laboratory, School of Biotechnology Jawaharlal Nehru University New Delhi 110 067 India

**Keywords:** betasatellite, chloroplast, geminivirus, oxygen‐evolving complex, plant defence, plant‐virus interactions, PsbP

## Abstract

Geminivirus disease complexes potentially interfere with plants physiology and cause disastrous effects on a wide range of economically important crops throughout the world. Diverse geminivirus betasatellite associations exacerbate the epidemic threat for global food security. Our previous study showed that βC1, the pathogenicity determinant of geminivirus betasatellites induce symptom development by disrupting the ultrastructure and function of chloroplasts. Here we explored the betasatellite‐virus‐chloroplast interaction in the scope of viral pathogenesis as well as plant defence responses, using *Nicotiana benthamiana*—Radish leaf curl betasatellite (RaLCB) as the model system. We have shown an interaction between RaLCB‐encoded βC1 and one of the extrinsic subunit proteins of oxygen‐evolving complex of photosystem II both *in vitro* and *in vivo*. Further, we demonstrate a novel function of the *Nicotiana benthamiana* oxygen‐evolving enhancer protein 2 (PsbP), in that it binds DNA, including geminivirus DNA. Transient silencing of *PsbP* in *N. benthamiana* plants enhances pathogenicity and viral DNA accumulation. Overexpression of PsbP impedes disease development during the early phase of infection, suggesting that PsbP is involved in generation of defence response during geminivirus infection. In addition, βC1‐PsbP interaction hampers non‐specific binding of PsbP to the geminivirus DNA. Our findings suggest that betasatellite‐encoded βC1 protein accomplishes counter‐defence by physical interaction with PsbP reducing the ability of PsbP to bind geminivirus DNA to establish infection.

## Introduction

Viruses as intracellular obligate parasites usurp the host machinery at each step of infection cycle such as replication, viral movement and pathogenesis (Whitham and Wang, 2004). To establish infection in permissive hosts, viruses take over highly orchestrated cellular events by interacting with various host factors. In addition, interactions with the specific host factor affect disease development, sustained systemic infection and host antiviral defence (Kong *et al*., 2014). Immune response of plants to viruses is mediated through complex host‐virus interactions that are often difficult to understand mechanistically. Considerable progress has been made in elucidating the plant defence response, and an increasing number of host factors that negatively regulate virus accumulation have been identified (Bhat *et al*., 2013).

The antiviral response against virus systemic infection is known to be significantly associated with chloroplast function (Bhattacharyya and Chakraborty, 2017; Bhattacharyya *et al*., 2015). Conversely, plant viruses primarily target and exploit chloroplasts to establish viral pathogenesis and symptom induction (Takahashi and Ehara, 1992). Various plant RNA virus encoded proteins interact with the proteins of chloroplastic machinery and polypeptides of the photosystem (Jang *et al*., 2013). The photosynthetic electron transport activity is hampered by RNA viruses such as *Tobacco mosaic virus* (TMV) and *Cucumber mosaic virus* (CMV).

Results from several studies have shown that photosystem II (PSII) exhibits antiviral response against various virus infections (Abbink *et al*., 2002; Jang *et al*., 2013). PSII inhibition with herbicide (3‐[3,4‐Dichlorophenyl]‐1,1‐dimethylurea) treatment and subsequent TMV infection leads to development of pronounced necrotic lesions and increased TMV accumulation (Abbink *et al*., 2002). CMV strain‐Y (CMV‐Y) infection has been reported to affect PSII activity by altering both the polypeptide composition of the oxygen‐evolving complex (OEC) and by decreasing the oxygen‐evolving activity (Takahashi and Ehara, 1992). The OEC present on the luminal side of PSII catalyzes the water‐splitting reaction (Debus, 1992; Wydrzynski and Satoh, 2005). In higher plants and green algae, the OEC encompasses three nuclear‐encoded extrinsic proteins; PsbO, PsbP and PsbQ (Miyao and Murata, 1985). PsbP is highly conserved in higher plants and is essential for core assembly and stability of PSII (Meierhoff and Westhoff, 1993; Yi *et al*., 2007). In *Nicotiana tabacum*, four isogenes code for the PsbP protein and are categorized as PsbP group‐I (isoform PsbP1A and PsbP5) and PsbP group‐II (isoform PsbP2FA and PsbP3F). Regardless of the group of PsbP isoform, all isoforms are collectively needed for optimal PSII activity (Ishihara *et al*., 2005). PsbQ protein modulates PSII activity by stabilizing the functional binding of PsbP to the OEC (Kakiuchi *et al*., 2012). Similarly, four PsbP isogenes of *N. benthamiana* encode NbPsbP1, NbPsbP2, NbPsbP3 and NbPsbP4, which show greater than 80% amino acid identity (Pérez‐Bueno *et al*., 2011).

PsbP, the regulator of photosynthetic oxygen evolution was found to be significantly decreased during CMV‐Y infection, whereas, PsbO, an essential component for oxygen evolution, remains unaltered. Changes in the polypeptide composition of OEC in the CMV‐infected *N. tabacum* plants seems to be associated with the molecular process of symptom induction (Takahashi and Ehara, 1992). *PsbO*‐silenced *N. benthamiana* plants are susceptible to several viruses and eventually leading to several‐fold increased accumulation of TMV, Alfalfa mosaic virus (AMV), and Potato virus X (PVX) (Abbink *et al*., 2002). Conversely, overexpression of PsbP reduces accumulation of AMV. The sequestration of the AMV coat protein (CP) by direct interaction of PsbP with CP mediates an antiviral response (Balasubramaniam *et al*., 2014). Silencing of *PsbP* enhances *Rice stripe virus* (RSV) and PVX induced symptom severity as well as virus load in *N. benthamiana* and *Oryza sativa*. Interaction between RSV‐encoded diseases specific protein and PsbP modulates symptom expression by disrupting the chloroplast structure and function (Kong *et al*., 2014).

Geminiviruses are non‐enveloped, circular single‐stranded DNA (ssDNA) viruses, which infect a wide range of plant species and cause considerable loss of agricultural productivity. The *Geminiviridae* family consists of nine genera, namely, *Becurtovirus, Begomovirus, Capulavirus, Curtovirus, Eragrovirus, Grablovirus, Mastrevirus, Topocuvirus* and *Turncurtovirus* (Zerbini *et al*., 2017). The genus *Begomovirus* comprises the most numerous and geographically widespread viruses that are transmitted by whitefly, *Bemisia tabaci* Genn. Begomoviruses have genomes that are either bipartite, with components known as DNA‐A and DNA‐B, or monopartite, with a genome that is homologue of the DNA‐A of bipartite viruses (Hanley‐Bowdoin *et al*., 2000). DNA‐A encodes for CP (AV1/V1), pre‐coat protein (AV2/V2), replication associated protein (Rep, AC1/C1), transcriptional activator protein (TrAP, AC2/C2), replication enhancer protein (REn, AC3/C3) and C4 protein (AC4/C4). DNA‐B encodes for nuclear shuttle protein (NSP, BV1) and movement protein (MP, BC1) (Zerbini *et al*., 2017). Most of the monopartite begomoviruses are associated with betasatellites (ssDNA satellite molecules) and/or alphasatellite (satellite‐like molecules) (Kumar *et al*., 2015; Mansoor *et al*., 2006; Nawaz‐ul‐Rehman and Fauquet, 2009; Saunders *et al*., 2000; Vinoth Kumar *et al*., 2017). Betasatellites are small circular single‐stranded DNA molecules of approximately 1.3 kb, associated with the majority of monopartite begomoviruses (Saunders *et al*., 2000). They depend upon their helper begomoviruses for replication and encapsidation. In some instances, betasatellite are essential for symptom induction and efficient viral pathogenesis (Briddon *et al*., 2001; Cui *et al*., 2004; Jose and Usha, 2003; Saunders *et al*., 2003, 2004).

Betasatellites typically contain a satellite conserved region (SCR), an adenine‐rich region and a single gene that encodes the βC1 protein (Jose and Usha, 2003; Saunders *et al*., 2004). SCR is a highly conserved region of 150 nt–200 nt that contains a potential hairpin loop structure comprising nonanucleotide TAATATTAC with similarity to the origin of replication of geminiviruses (Briddon *et al*., 2003). The multitasking βC1 protein functions as a major symptom determinant (Bhattacharyya *et al*., 2015; Yang *et al*., 2008), and suppressor of gene silencing (Cui *et al*., 2005; Li *et al*., 2014a). βC1 protein also attenuate the host proteasomal machinery (Eini *et al*., 2009; Shen *et al*., 2011, 2016), and suppress the jasmonic acid (JA) response (Li *et al*., 2014b; Yang *et al*., 2008).

Our previous study provided the initial evidence of chloroplast localization of a DNA virus encoded protein, which affected photosynthesis (Bhattacharyya *et al*., 2015). Radish leaf curl betasatellite (RaLCB)‐encoded βC1 protein is localized into the chloroplasts of the infected *N. benthamiana* plants and causes damage to the OEC of PSII. The veinal chlorosis symptom induction by betasatellite infection was associated with βC1‐mediated disruption of the chloroplast structure and function. However, the mechanistic interaction between βC1 and chloroplast protein(s), and the resulting effect on the disease development remains undetermined. In this present study, we further explored the virus‐chloroplast interaction in the scope of viral pathogenesis as well as plant defence response. Here, we identified interaction of RaLCB‐encoded βC1 protein with the OEC protein subunit 2, PsbP and decreased DNA binding activity of PsbP. Interestingly, PsbP expression was negatively correlated with disease development. Further, the study demonstrates that βC1, the pathogenicity determinant of betasatellites, overcomes the PsbP‐mediated defence response during the later phases of geminivirus infection.

## Results

### RaLCB‐encoded **β**C1 protein interacts with host PsbP

**Figure 1:**
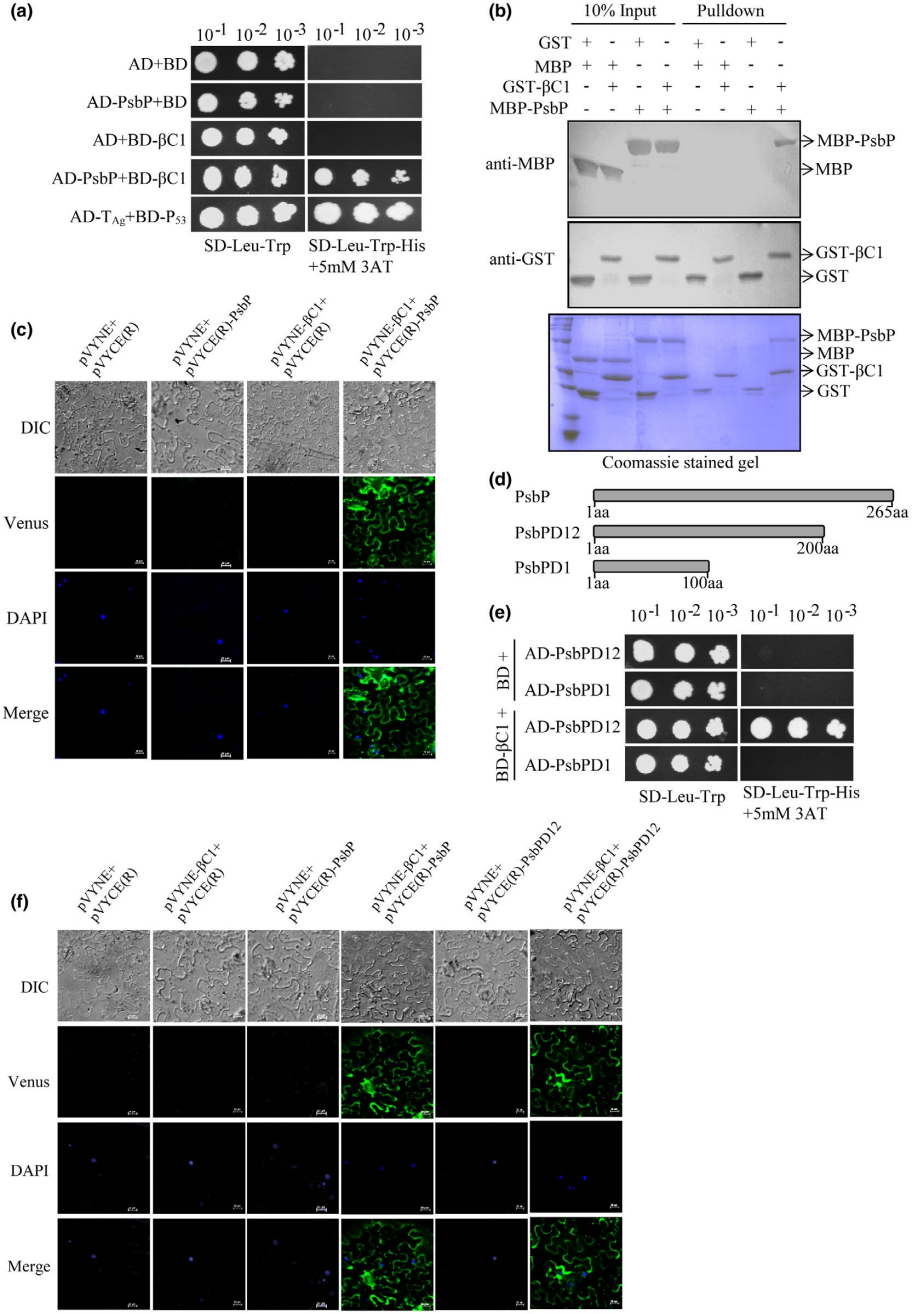
The βC1 protein of the Radish leaf curl betasatellite (RaLCB) interacts with the nuclear‐encoded chloroplast protein; PsbP. (a) Yeast strain AH109 cells were co‐transformed with different combinations of yeast two‐hybrid constructs as mentioned. The co‐transformed AH109 cells were grown on either synthetic dropout medium SD‐Leu‐Trp or selection medium SD‐Leu‐Trp‐His supplemented with 5 mM 3‐amino‐1,2,4‐triazole as serial dilution from cultures OD_600 _of 1.0. Yeast cells co‐transformed with AD plus BD or AD‐T_Ag _plus BD‐P_53 _act as negative and positive controls, respectively. (b) An equal amount of purified GST/GST‐βC1 protein was used to pull‐down MBP/MBP‐PsbP protein. Immunoblotting was carried out using either anti‐GST or anti‐MBP antibodies. Coomassie‐stained gel served to monitor the amount of protein present in input and pull‐down. (c) Different combinations of *Agrobacterium* cells harbouring bimolecular fluorescence complementation (BiFC) constructs were co‐infiltrated into the leaves of 3 to 4 weeks old *N. benthamiana* plants as mentioned. The lower epidermal cells of co‐infiltrated leaves were visualized under a confocal microscope. The reconstituted Venus protein fluorescence was observed in the periphery of the epidermal cell of leaves co‐infiltrated with *Agrobacterium* harbouring both pVYNE‐βC1 and pVYCE(R)‐PsbP constructs. Nuclei of the epidermal cells were stained with 4, 6‐diamidino‐2‐phenylindole (DAPI). Scale bar represents 20 μM. (d) Schematic representation of the C‐terminal deletion constructs of PsbP protein used for yeast two‐hybrid assay and BiFC assay. (e) Yeast two‐hybrid assay carried out with βC1 and different domains of PsbP. (f) BiFC assay was performed on *N. benthamiana* plants leaves infiltrated with *Agrobacterium* carrying plasmid combinations as mentioned. Scale bar represents 20 μm.

Betasatellite infections, as well as transient expression of βC1 protein, intrude plant’s physiology by causing structural and functional damage to chloroplast (Bhattacharyya *et al*., 2015). To explore the molecular basis of βC1‐induced chloroplast damage we identified a *N. benthamiana*‐encoded protein interacting with βC1 by immunoprecipitation assay. The 23 kDa protein that immunoprecipitated with the βC1 protein was found to be a *N. benthamiana* chloroplastic photosynthetic oxygen‐evolving protein 23 kDa subunit, PsbP1 by sequencing through MALDI‐TOF MS analysis (Fig. S1a). We confirmed the interaction between βC1 and NbPsbP1 (referred here as PsbP) by yeast two‐hybrid analyses. Yeast transformants carrying BD‐βC1 and AD‐PsbP plasmids were able to grow on SD‐Leu‐Trp‐His selection plates supplemented with 5 mM 3‐amino‐1,2,4‐triazole, whereas the yeast transformants carrying negative control plasmids (AD and BD, AD‐PsbP and BD or AD and BD‐βC1) were unable to grow (Fig. 1a). Similarly, yeast transformants carrying positive control plasmids AD‐T_Ag_ and BD‐P_53_ grew on selection plates (Fig. 1a). Similarly, we confirmed the interaction between βC1 and PsbP encoded by *N. tabacum* Samsun NN (referred here as NtPsbP) by yeast two‐hybrid analysis (Fig. S1b). These results confirmed interaction of βC1 with PsbP proteins in yeast cells.

*In vitro* GST pull‐down assays were performed to confirm the direct interaction between βC1 and PsbP. The results showed that MBP‐PsbP was able to pull‐down GST‐βC1 but not with GST. In addition, the MBP protein was unable to pull‐down either GST‐βC1 or GST. These results indicate specific interaction between βC1 and PsbP *in vitro* (Fig. 1b). Further, we carried out bimolecular fluorescence complementation (BiFC) assays in *N. benthamiana* plants using BiFC vectors, pVYNE and pVYCE(R) encoding the N‐terminal and C‐terminal sequences of the Venus fluorescent protein, respectively. Venus fluorescent protein is a variant of Yellow fluorescent protein (YFP), which exhibits brighter fluorescence than YFP and Cyan fluorescent protein (Waadt *et al*., 2008). The reconstituted Venus fluorescence was observed in the epidermal cells of leaves co‐infiltrated with *Agrobacterium* carrying pVYNE‐βC1 and pVYCE(R)‐PsbP constructs (Fig. 1c). However, no fluorescence was observed in the epidermal cells of leaves co‐infiltrated with pVYNE and pVYCE(R), pVYNE and pVYCE(R)‐PsbP or pVYNE‐βC1 and pVYCE(R) (Fig. 1c). The nuclei of the epidermal cells were detected by 4, 6‐diamidino‐2‐phenylindole (DAPI) staining.

To identify the region of PsbP that interacts with βC1, yeast two‐hybrid analyses were carried out with C‐terminal deletion constructs PsbPD12 and PsbPD1 for the expression of amino acids 1‐200 and 1‐100, respectively of the PsbP protein (Fig. 1d). The results indicated that βC1 interacts with PsbPD12 but not with PsbPD1 (Fig. 1e). The interaction of βC1 with PsbPD12 was further validated by BiFC assay in *N. benthamiana.* The fluorescence was observed in the epidermal cells of leaves co‐infiltrated with pVYNE‐βC1 plus pVYCE(R)‐PsbP and pVYNE‐βC1 plus pVYCE(R)‐PsbPD12 indicating the interaction between βC1 and PsbPD12 *in planta* (Fig. 1f). Taken together, these results demonstrated that βC1 interacts with PsbPD12 in the epidermal cells of *N. benthamiana*.

### PsbP binds to RaLCB DNA *in vitro*

**Figure 2:**
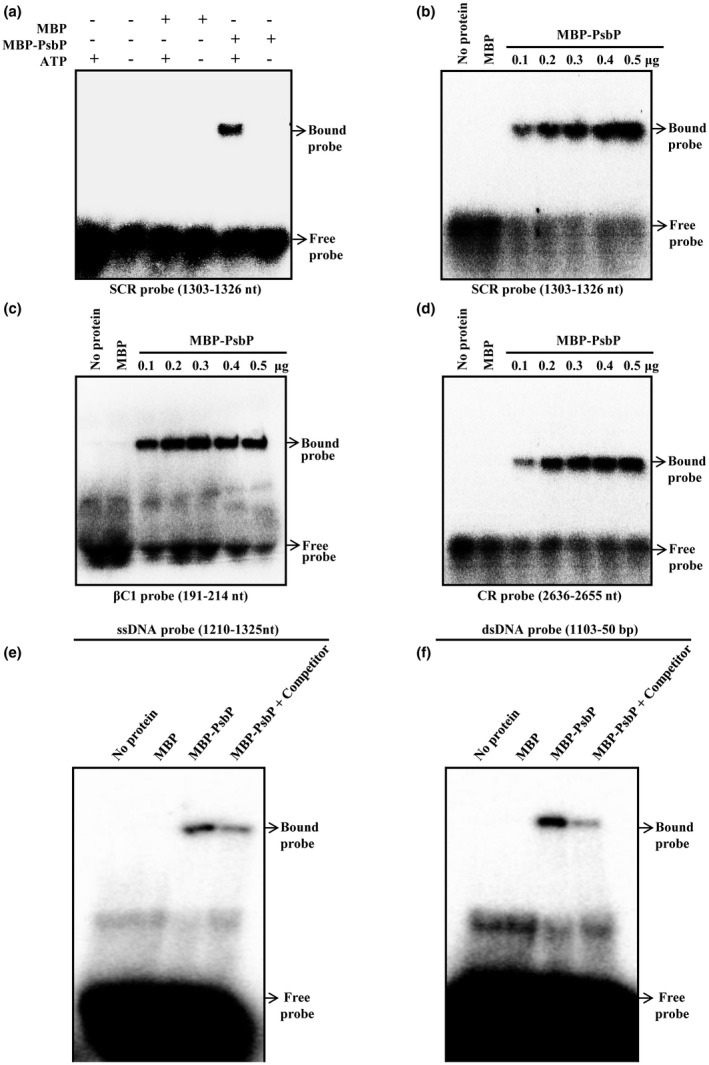
PsbP protein binds to geminivirus DNA *in vitro*. The autoradiograph of electrophoretic mobility shift assay (EMSA) showing purified MBP‐PsbP protein binding to the SCR region probe in the presence of ATP (a, b), the βC1 region probe (c) of betasatellite and the CR region probe (d) of helper geminivirus DNA‐A. Autoradiograph of EMSA showing binding of PsbP protein to single‐stranded DNA (ssDNA) (e), and double‐stranded (dsDNA) (f) fragment of betasatellite genome in the presence of competitor DNA (either NbActin ssDNA or pMAL‐c2X plasmid). Location of probe into the geminivirus and betasatellite DNA is indicated for each autoradiograph. The purified MBP protein was used as negative control.

To elucidate the role of PsbP in plant‐geminivirus interaction, we investigated functionality of the protein by in silico analysis. Prediction by helix‐turn‐helix motif tool shows the presence of helix‐turn‐helix motif at amino acid position 39 to 60, indicating a possible DNA binding feature of the PsbP protein. To confirm the ability of PsbP to bind RaLCB DNA *in vitro*, we performed electrophoretic mobility shift assay (EMSA) using the purified recombinant MBP‐PsbP protein and DNA fragments corresponding to the SCR region of the betasatellite. EMSA results showed that MBP‐PsbP binds to DNA in the presence of ATP (Fig. 2a) and the DNA binding activity of PsbP was found to increase with the increasing concentration of ATP (Fig. S2a). PsbP protein also showed binding to ssDNA corresponding to SCR (Fig. 2b) and βC1 (Fig. 2c) regions of betasatellite as well as to the CR region (Fig. 2d) of helper virus DNA. Moreover, a ssDNA probe located at positions 1210 to 1325 in the betasatellite genome, and a double‐stranded DNA (dsDNA) probe located at position 1103 to 50 in the betasatellite genome, were also used for EMSA assays. Results showed that MBP‐PsbP protein can bind to both ssDNA (Fig. 2e) and dsDNA (Fig. 2f) corresponding to betasatellite genome, while MBP protein used as negative control did not bind to either ssDNA or dsDNA. Addition of 100‐fold molar excesses of ssDNA competitor (NbActin ssDNA oligo) (Fig. 2e, lane 4) competed for binding with PsbP. Likewise, 100‐fold molar excesses of dsDNA competitor (pMAL‐c2X plasmid DNA) (Fig. 2f, lane 4) also competed for binding with PsbP. Together, these results indicate that *in vitro* PsbP binding to ssDNA and dsDNA is sequence non‐specific.

### **β**C1‐PsbP interaction interferes with the ability of PsbP to bind viral DNA

**Figure 3:**
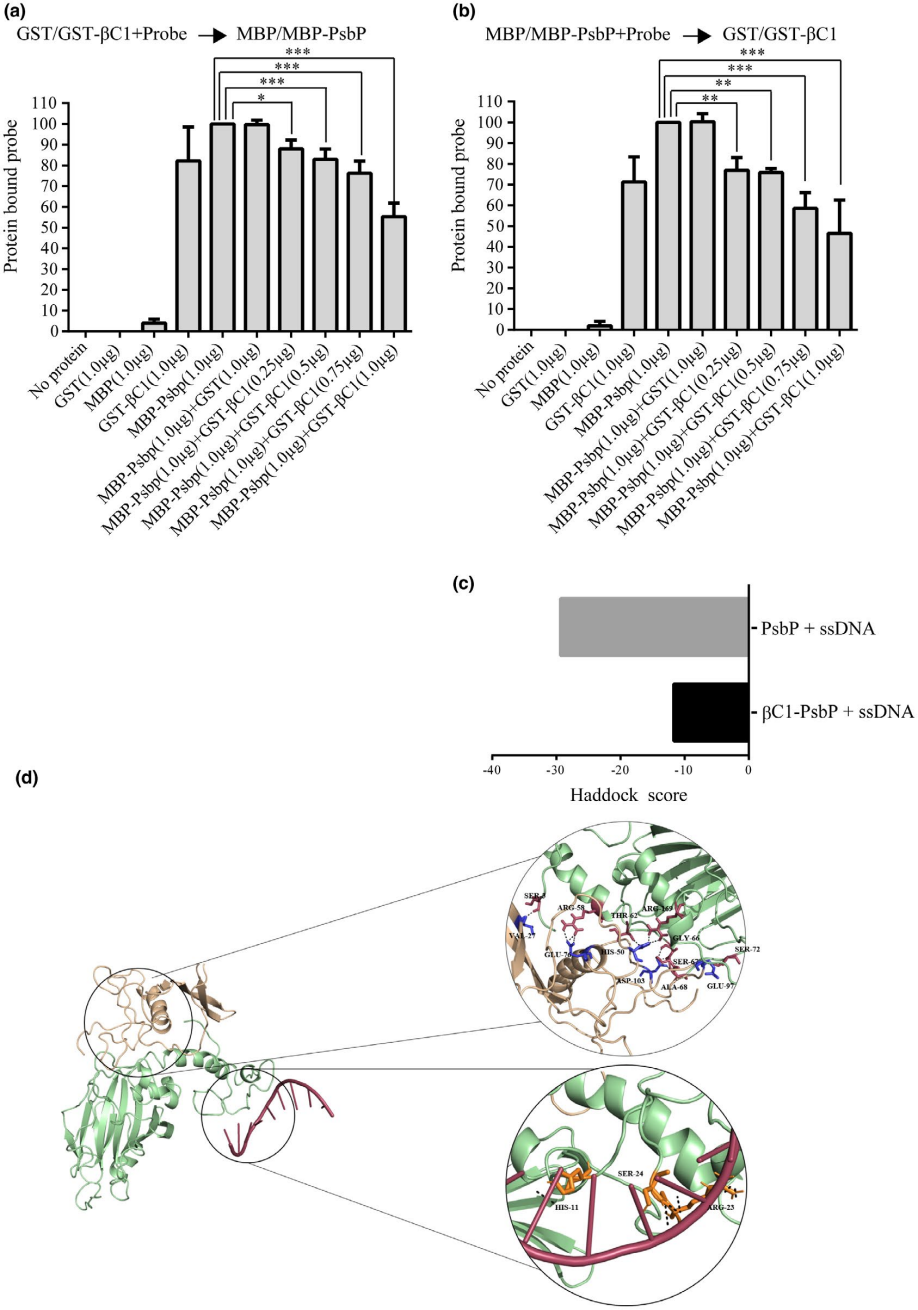
βC1 interferes with PsbP binding to viral DNA. (a) Protein competitive EMSA experiment was performed by incubating MBP‐PsbP protein with DNA‐probe pre‐incubated with increasing concentration of GST‐βC1 protein. (b) Protein competitive EMSA experiment was performed by addition of increasing concentration of GST‐βC1 protein to DNA‐probe pre‐incubated with MBP‐PsbP protein. Purified GST protein was used as negative control. The 32p‐labelled single‐stranded oligonucleotide substrate from the SCR region of betasatellite (1303 nt–1326 nt) was used as DNA‐probe. The amount of protein bound DNA was quantified using the ImageJ software and plotted using the Graph Pad Prism 6 software. Asterisks indicate reactions in which amount of protein bound DNA was significantly decreased compared with the amount of protein bound DNA in the reaction with PsbP (considered as 100%). (*, *P* < 0.05; **, *P* < 0.01; ***, *P* < 0.001) as determined by Dunnett's multiple comparisons test by analysis of variance (ANOVA). The values are mean of the per cent of protein bound DNA from three independent experiments. (c) *In silico* docking of ssDNA (located at positions 1303 to 1326 in the betasatellite genome) with PsbP and PsbP‐βC1 complex. (d) *In silico* docking to identify potential residues of PsbP that interacts with either βC1 protein or ssDNA.

Since PsbP can bind to DNA and also interacts with βC1 protein, we tested whether the βC1‐PsbP interaction affects DNA binding ability of PsbP. Previous studies showed that βC1 protein can bind to DNA *in vitro* (Cui *et al*., 2005) and forms a multimeric complex in *in vitro* conditions (Cheng *et al*., 2011). The results of EMSA performed with purified recombinant GST‐βC1 protein confirms the DNA binding ability of βC1 protein (Fig. S2b). It is important to note that βC1‐DNA complex migrate at the same rate as PsbP‐DNA complex electrophoresed on 3.7% native polyacrylamide gel, probably due to similar electrostatic properties of those complexes (Fig. S3a,b). To study the effect of βC1 on the DNA binding activity of PsbP, we analysed the PsbP‐DNA binding ability with increasing concentration of purified recombinant GST‐βC1 by competition experiment. For this purpose, MBP/MBP‐PsbP protein was added to GST/GST‐βC1 protein pre‐incubated with radiolabelled DNA‐probe or *vice versa*. Although, EMSA results could not differentiate PsbP‐DNA and βC1‐DNA complexes, the amount of DNA‐protein complex was found to be decreased with increasing concentration of βC1 protein (Fig. S3a,b). The protein competitive EMSA autoradiographs were quantified using the ImageJ software and plotted (Fig. 3a,b). These results indicate that the equilibrium towards βC1‐PsbP interaction is favoured rather than βC1 and/or PsbP‐DNA interactions.

The 3D structures of PsbP and βC1 proteins were not available in Protein Data Bank (PDB) and therefore, we have used Phyre2 to predict 3D structures of these proteins. The MD simulated structure showed stable conformation of PsbP and βC1. The complex of PsbP‐βC1 was generated using HADDOCK, which suggest that PsbP amino acid residues corresponding to 57‐72 and 141‐172 are involved in strong interaction with βC1 (Fig. S4a). After each successful docking result, the structures are clustered and the resulting clusters are ranked according to the HADDOCK score. The best docking solution is considered based on the docking scores. Further, hydrogen bond analyses were carried out to compare the different docking complexes. Additionally, we predicted the region encompassing amino acid residues 17‐42 as potential DNA binding sites in PsbP and the crucial ssDNA binding residues were highlighted (Fig. S4b). We predicted a stronger interaction between ssDNA and PsbP as compared to that of ssDNA with PsbP‐βC1 complex (Fig. 3c). Similarly, while docking with dsDNA we predicted stronger interaction between dsDNA and PsbP as compared to that of dsDNA with PsbP‐βC1 complex (Fig. S4c,d,e). Thus, βC1 probably interacts with the α‐helix (amino acids 36‐60) region of PsbP and interferes with its DNA binding ability (Figs 3d and S4e).

### Silencing of *PsbP* enhances viral pathogenesis in *N. benthamiana* plants

**Figure 4:**
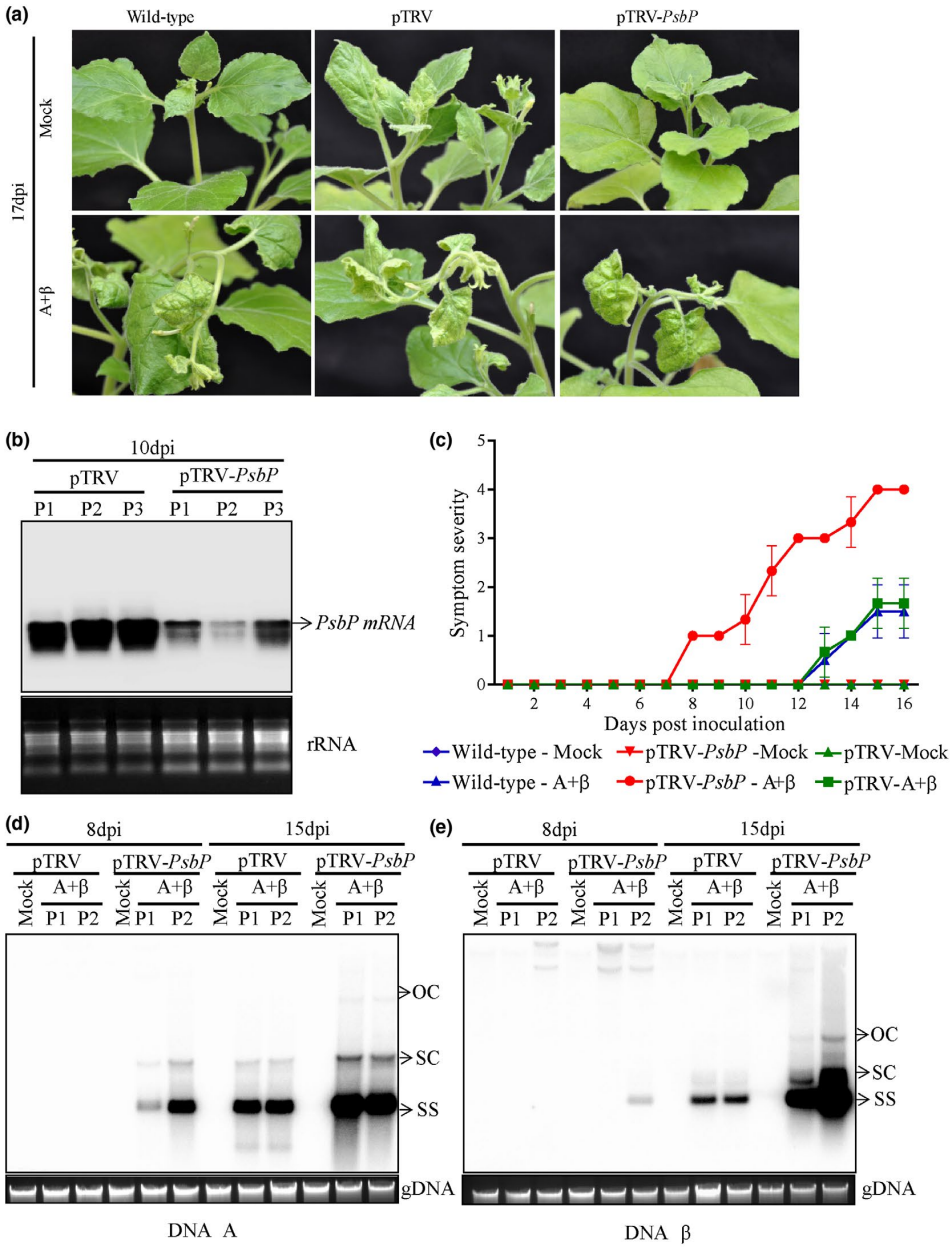
Silencing of *PsbP* accelerates disease development in *N. benthamiana*. (a) Betasatellite induced symptoms on wild‐type, *PsbP*‐silenced or pTRV‐infiltrated plants at 17 days post‐inoculation (dpi). (b) Comparative level of *PsbP* transcripts in *PsbP*‐silenced or pTRV‐infiltrated *N. benthamiana* plants detected by northern blotting analysis at 10 days post‐infiltration of silencing constructs. Ethidium bromide stained gels showing ribosomal RNA (rRNA) served as loading control. P1, P2 and P3 indicate samples from three independent plants. (c) Appearance of RaLCB‐mediated symptoms at different dpi on wild‐type*, PsbP*‐silenced and pTRV‐infiltrated plants. Southern blotting analysis indicating comparative level of DNA‐A (d) and DNA β (e) in either *PsbP*‐silenced or pTRV‐infiltrated *N. benthamiana* plants. The ethidium bromide stained gel showing plant genomic DNA (gDNA) served as loading control. P1 and P2 indicate samples from two independent plants. Viral DNA forms are indicated as OC (open circular), SC (supercoiled) and SS (single stranded).

Transient silencing of the *PsbP* gene was achieved in *N. benthamiana* plants using the pTRV‐*PsbP*‐silencing construct, while pTRV‐infiltrated plants served as the control. The leaves of *PsbP*‐silenced plants remained yellowish green as compared to the pTRV‐control plants (Figs 4a and S5a,b). Transcript level of *PsbP* in systemic leaves of *PsbP*‐silenced and pTRV‐control plants at 10 days post‐inoculation (dpi) was measured by RNA blot analysis. Silencing of the *PsbP* gene was confirmed by detection of marked reduction of *PsbP* mRNA in the pTRV‐*PsbP* infiltrated plants as compared to pTRV‐control plants (Fig. 4b).

To decipher the role of PsbP during geminivirus infection, we co‐inoculated the *PsbP*‐silenced and pTRV‐control *N. benthamiana* plants with *Tomato leaf curl New Delhi virus* (ToLCNDV) DNA‐A (A) and RaLCB (β). A + β‐inoculated wild‐type and pTRV‐control plants showed downward leaf curling symptom at around 13 dpi, while symptom appearance was noticed much earlier (at 8 dpi) on *PsbP*‐silenced plants (Figs 4a,c and S5b,c; Supplementary Table S1). The accumulation of viral DNA in the systemic leaves of betasatellite infected *PsbP*‐silenced plants was found to be significantly higher than the pTRV‐control plants (Fig. 4d). Similarly, *PsbP*‐silenced plants showed higher accumulation of helper virus DNA at 8 dpi as well as 15 dpi compared to that of pTRV‐control plants (Fig. 4e; Supplementary Table S1). These results revealed that transient silencing of *PsbP* greatly enhances the symptom induction and geminivirus DNA accumulation, indicating that PsbP plays an antiviral role during geminivirus infection.

### Overexpression of PsbP delays disease development and reduces viral DNA accumulation during betasatellite infection

**Figure 5:**
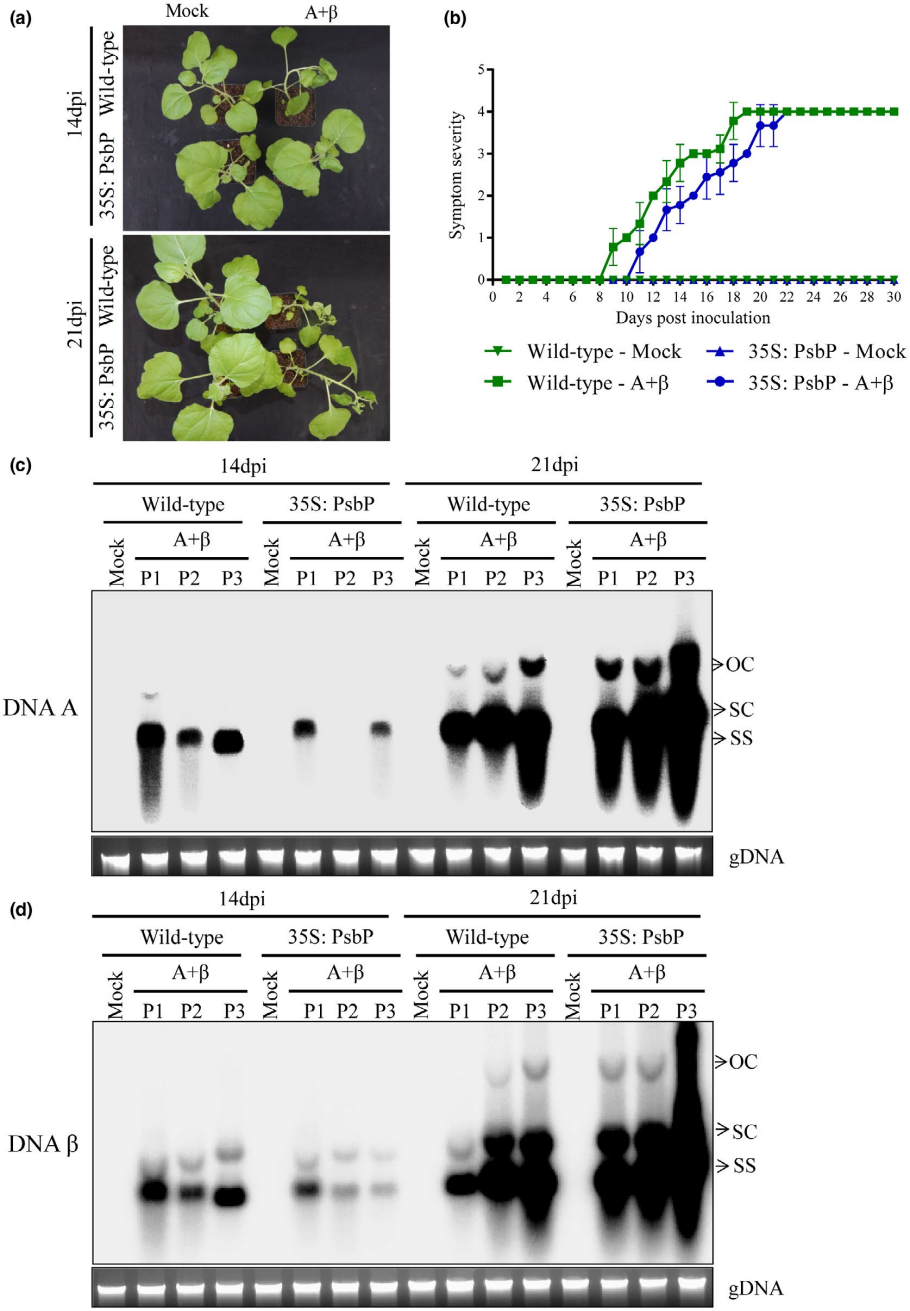
PsbP overexpression interferes with symptom induction and viral DNA accumulation during betasatellite infection. (a) PsbP overexpressing (35: PsbP) and wild‐type *N. benthamiana* plants exhibiting symptom induction following betasatellite infection. (b) Appearance of betasatellite‐mediated symptoms on wild‐type and PsbP overexpressing transgenic *N. benthamiana* plants. Southern blotting analysis indicating comparative level of DNA‐A (c) and DNA β (d) in wild‐type and PsbP overexpressing transgenic *N. benthamiana* plants. P1, P2 and P3 indicate samples from three independent plants. Viral DNA forms are indicated as OC (open circular), SC (supercoiled) and SS (single stranded). Ethidium bromide stained gel showing plant genomic DNA (gDNA) serves as loading control.

To demonstrate the critical role of PsbP during betasatellite infection, we co‐inoculated wild‐type and 35S‐PsbP transgenic *N. benthamiana* plants with A + β. Wild‐type plants inoculated with A + β showed downward leaf curling at 9 dpi and developed severe vein clearing symptom at 14 dpi. However, the betasatellite‐mediated symptom induction was found to be delayed (by 2 days) on the 35S‐PsbP transgenic plants compared with A + β inoculated wild‐type plants (Fig. 5a,b; Supplementary Table S2). Furthermore, the accumulation of both viral genome and betasatellite DNA was assessed by Southern blotting analysis. At 14 dpi, accumulation of both helper virus and betasatellite DNA was found to be reduced in 35S‐PsbP transgenic plants compared to that of the wild‐type plants. On the contrary, at 21 dpi the level of helper virus and betasatellite DNA is comparable in A + β co‐inoculated wild‐type and 35S‐PsbP transgenic *N. benthamiana* plants (Fig. 5c,d; Supplementary Table S2). Taken together, these results suggest that PsbP‐mediated defence response delays symptom induction and reduces viral titre during the early stage of geminivirus infection. However, the PsbP‐mediated defence response can be attenuated by the virus during the later stage of infection.

### PsbP isoforms contribute to defence against geminivirus

**Figure 6:**
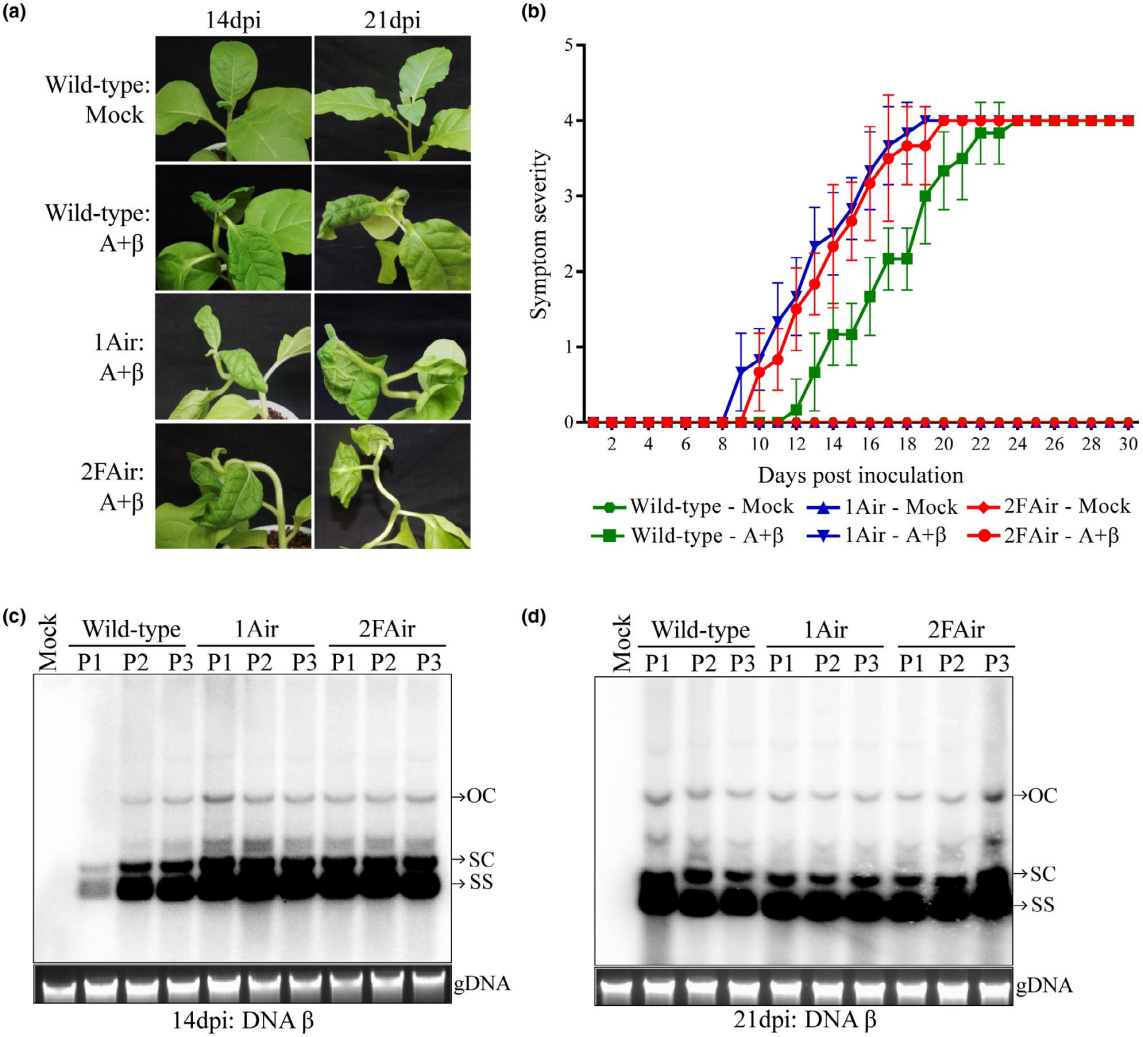
PsbP‐mediated impediment against geminivirus infection depends on overall expression of *PsbP* isoforms. (a) Betasatellite induced symptoms on wild‐type and transgenic *PsbP* dRNAi (1Air and 2FAir) *N. tabacum* plants. (b) Symptom severity graph shows appearance of betasatellite‐mediated symptoms on wild‐type, 1Air and 2Fair *N. tabacum* plants at different dpi. Southern blotting analysis shows comparative level of DNA β at 14 dpi (c) and 21 dpi (d) in wild‐type, 1Air, and 2FAir *N. tabacum* plants. P1, P2 and P3 denote samples from three independent plants. Viral DNA forms are indicated as OC (open circular), SC (supercoiled) and SS (single stranded). Ethidium bromide stained gel showing plant genomic DNA (gDNA) serves as loading control.

An earlier study reported that, in PsbP 1Air lines, two isoforms (PsbP1A and PsbP5B) were down‐regulated, whereas, in PsbP 2FAir lines, three isoforms (PsbP2AF, PsbP3F and PsbP5B) were down‐regulated (Ishihara *et al*., 2005). Although the photosynthetic function of PsbP is known to be dependent on the total amount of PsbP protein irrespective of its isoform type, the role of different PsbPs in plant defence remain elusive. To explore the function of different PsbP isoforms in the perspective of plant defence against geminiviruses, we inoculated the wild‐type, and transgenic *PsbP* dRNAi (IAir and 2FAir) *Nicotiana tabacum* Samsun NN plants with A + β. Upon co‐inoculation with A + β, the wild‐type, IAir and 2FAir lines showed downward leaf curling, mild vein clearing, and stem bending (Fig. 6a). However, symptoms on transgenic *PsbP* dRNAi tobacco lines appeared earlier (by 2 days to 3 days) as compared to inoculated wild‐type tobacco plants (Fig. 6b; Supplementary Table S3). In addition, higher accumulation of betasatellite DNA was found at 14 dpi in IAir and 2FAir lines as compared to the wild‐type plants (Fig. 6c). However, at 21 dpi, the betasatellite accumulation in wild‐type plants showed minor reduction as compared to IAir and 2FAir lines (Fig. 6d). These observations correlate with the results obtained from the earlier experiments and conjointly confirm that effect of PsbP‐mediated defence response is diminished during the later stage of virus infection. Likewise, accumulation of helper virus DNA in dRNAi lines (in IAir and 2FAir) at 14 dpi was found to be higher than in wild‐type plants (Fig. S6a), while the helper virus DNA accumulations at 21 dpi were only slightly altered in wild‐type plants compared to that of *PsbP* dRNAi lines (Fig. S6b). These results are consistent with the negative correlation of the PsbP protein accumulation with betasatellite‐mediated disease development. These results suggest that both PsbP group‐I and PsbP group‐II isoforms contribute to PsbP‐mediated antiviral state against geminivirus in tobacco.

Betasatellite infections affect the photosynthetic function and disrupt the ultrastructure of chloroplast structure in *N. benthamiana* plants (Bhattacharyya *et al*., 2015). In comparison to A + β‐infected wild‐type plants, the photosynthetic activity of PSII was found to be reduced in either mock or A + β‐inoculated transgenic 1Air and 2FAir lines (Fig. S7a). However, the chloroplasts of mock‐inoculated transgenic 1Air and 2FAir lines remain largely unaltered while the chloroplasts from A + β‐infected wild‐type plants show severe ultrastructural damage (Fig. S7b). Taken together, these results demonstrate that mere reduction of photosynthetic activity of PSII by modulating the PsbP is not sufficient to cause the ultrastructural damage of chloroplast.

## Discussion

Geminivirus disease complexes cause disastrous effects on a wide range of economically important crops throughout the world. The severe epidemic threat for the crops aroused since the betasatellite associated with the geminivirus disease complex could be trans‐replicated by diverse helper begomoviruses. Plants have evolved diverse defence responses against geminivirus infection by instigating ubiquitin/26S proteasome system, RNA silencing machinery and numerous host cellular factors associated with innate immunity (Cui *et al*., 2005; Eini *et al*., 2009; Kushwaha *et al*., 2015; Li *et al*., 2014a; Sahu *et al*., 2016; Shen *et al*., 2011, 2016). In an earlier study, we reported that RaLCB in association with ToLCNDV DNA‐A induces severe downward leaf curling, veinal chlorosis and stunting of *N. benthamiana* plants (Singh *et al*., 2012).

Systemic infection of RNA viruses often induces chlorosis and mosaic pattern on the leaves of infected plants. Although mechanism of systemic symptom expression remains elusive, several studies associate the viral symptom induction with deterioration of pigment composition and chloroplast structure and function (Bhattacharyya *et al*., 2015; Hodgson *et al*., 1989; Takahashi and Ehara, 1992). TMV inhibits electron transport rate of PSII but not PSI in the infected leaves of spinach. The association of TMV encoded CP with PSII complex contributes to the reduced Fv/Fm ratio in the infected leaves (Hodgson *et al*., 1989; Reinero and Beachy, 1989). Similarly, CMV infection significantly reduces the level of the PsbP polypeptide in OEC, oxygen evolution by OEC and electron transport rate of PSII in the infected tobacco leaves, whereas the level of PsbO polypeptide of OEC remains largely unaltered (Reinero and Beachy, 1989). Similar to plant RNA viruses, betasatellite infection alters the composition of the extrinsic protein of OEC, drastically reduces the number of active reaction centres and subsequently affects the electron transfer rate of PSII. Betasatellite infection down‐regulates the expression of genes involved in chlorophyll biosynthesis, plastid translocation and chloroplast development (Bhattacharyya *et al*., 2015).

In addition to the photosynthetic function of PsbP regulating OEC, its role in plant defence response has recently been recognized. Earlier studies on plant RNA viruses indicate PsbP‐mediated antiviral defence response that remains elusive at the molecular level (Balasubramaniam *et al*., 2014; Kong *et al*., 2014; Takahashi and Ehara, 1992). Silencing of *PsbP* also causes increased symptom expression and viral accumulation in plants infected with AMV and RSV (Balasubramaniam *et al*., 2014; Kong *et al*., 2014). The current study showed *PsbP*‐silencing resulted in enhanced disease development on infected *N. benthamiana* plants (Fig. 4). Following infection, the transgenic *PsbP* dRNAi tobacco plants showed earlier symptom induction and enhanced viral DNA accumulation in comparison to the wild‐type plants (Fig. 6). Correspondingly, overexpression of PsbP delays symptom induction and reduces viral DNA accumulation during the initial phase of disease progression. In contrast, overexpression of PsbP in *N. benthamiana* does not affect either symptom expression or the accumulation of viral DNA during later stage of infection (Fig. 5). These results suggest that geminiviruses mitigate the PsbP‐mediated antiviral defence response in order to establish pathogenesis.

The movement protein of a bipartite geminivirus (*Abutilon mosaic virus*, AbMV) interacts with nuclear‐encoded and chloroplast localized heat shock cognate 70 kDa protein (Krenz *et al*., 2010). Bipartite geminivirus (AbMV) as well as betasatellite (RaLCB) infection affect the structure of chloroplasts (Bhattacharyya *et al*., 2015; Krenz *et al*., 2012; Schuchalter‐Eicke and Jeske, 1983). PsbP, a nuclear‐encoded component of OEC of PSII localizes into the chloroplast and cytoplasm (Balasubramaniam *et al*., 2014; Kong *et al*., 2014). We have previously demonstrated that RaLCB‐βC1 localizes into the nucleus and as well as chloroplast (Bhattacharyya *et al*., 2015). In this study, for the first time, we demonstrate the interaction between geminivirus betasatellite encoded protein and PsbP. Although geminiviruses are known to replicate in the nucleus of the infected cells and migrate to the cytoplasm, the virions as well as viral genome, have also been detected in chloroplasts and other plastids (Groning *et al*., 1987). EMSA results showed binding of PsbP to geminivirus as well as betasatellite DNA (Figs 2 and S2) in a sequence non‐specific manner. DNA binding activity of PsbP may have additional role in host biology, which needs further investigation. Together, it appears that chloroplast and/or cytoplasm serve as the possible site(s) where βC1‐PsbP and PsbP‐DNA interaction might occur. However, further investigations are required to ascertain precise site(s) of these interactions.

Various studies demonstrate the role of βC1 in mediating viral counter‐defence to suppress plant defence response. The interaction of TYLCCNB‐βC1 with ASYMMETRIC LEAVES1 (AS1) in the molecular disguise of ASYMMETRIC LEAVES2 (AS2) selectively suppresses the JA response (Yang *et al*., 2008). TYLCCNB‐βC1 interaction with S‐adenosyl homocysteine hydrolase (SAHH) limits the methyl group donor for methylation by inhibiting the SAHH activity. Thus, either betasatellite infection or only transient expression of TYLCCNB‐βC1 protein reduces both host and viral genome methylation globally and stabilizes geminivirus disease complex (Yang *et al*., 2011). The βC1 protein interacts with MYC2 transcription factor and interferes with dimerization of MYC2 essential for JA‐mediated defence response in *Arabidopsis thaliana* (Li *et al*., 2014b).

Plant RNA viruses pre‐empt the PsbP‐mediated antiviral state by deploying viral protein that interact with the chloroplast‐targeted PsbP protein in the cytosol. The CP of AMV and diseases specific protein of RSV interact with PsbP in the cytosol, thereby modulating symptom induction and viral pathogenesis (Balasubramaniam *et al*., 2014; Kong *et al*., 2014). The βC1‐PsbP interaction possibly blocks the PsbP‐role in the electron transport of PSII; this interaction might block the PSII dependent pathogen restriction too. To overcome the PsbP‐mediated antiviral state, the βC1 protein interacts and interferes with PsbP binding to geminivirus DNA.

Our previous study demonstrates that either betasatellite infection or transient expression of the βC1 protein disrupts the electron transport rate by PSII and damage the photosynthetic machinery. Studies on plant RNA viruses show evidence that interruption of the PSII activity by viral proteins leads to the suppression of defence response, defence‐associated reactive oxygen species (ROS) production, and programmed cell death (Balasubramaniam *et al*., 2014; Kong *et al*., 2014; Takahashi and Ehara, 1992). The ability of PsbP to interact with geminivirus DNA leads to delayed symptom induction and reduced viral DNA accumulation in PsbP overexpressing *N. benthamiana* plants. On the contrary, overexpression of PsbP could not effectively eradicate the virus infection suggesting ability of the virus to overcome PsbP‐mediated defence. To understand the significance of βC1‐PsbP interaction in virus infection, we analysed the DNA binding ability of PsbP in the presence of increasing concentration of the βC1 protein. Our competitive EMSA result shows that the βC1‐PsbP interaction interferes with the DNA binding ability of PsbP.

**Figure 7:**
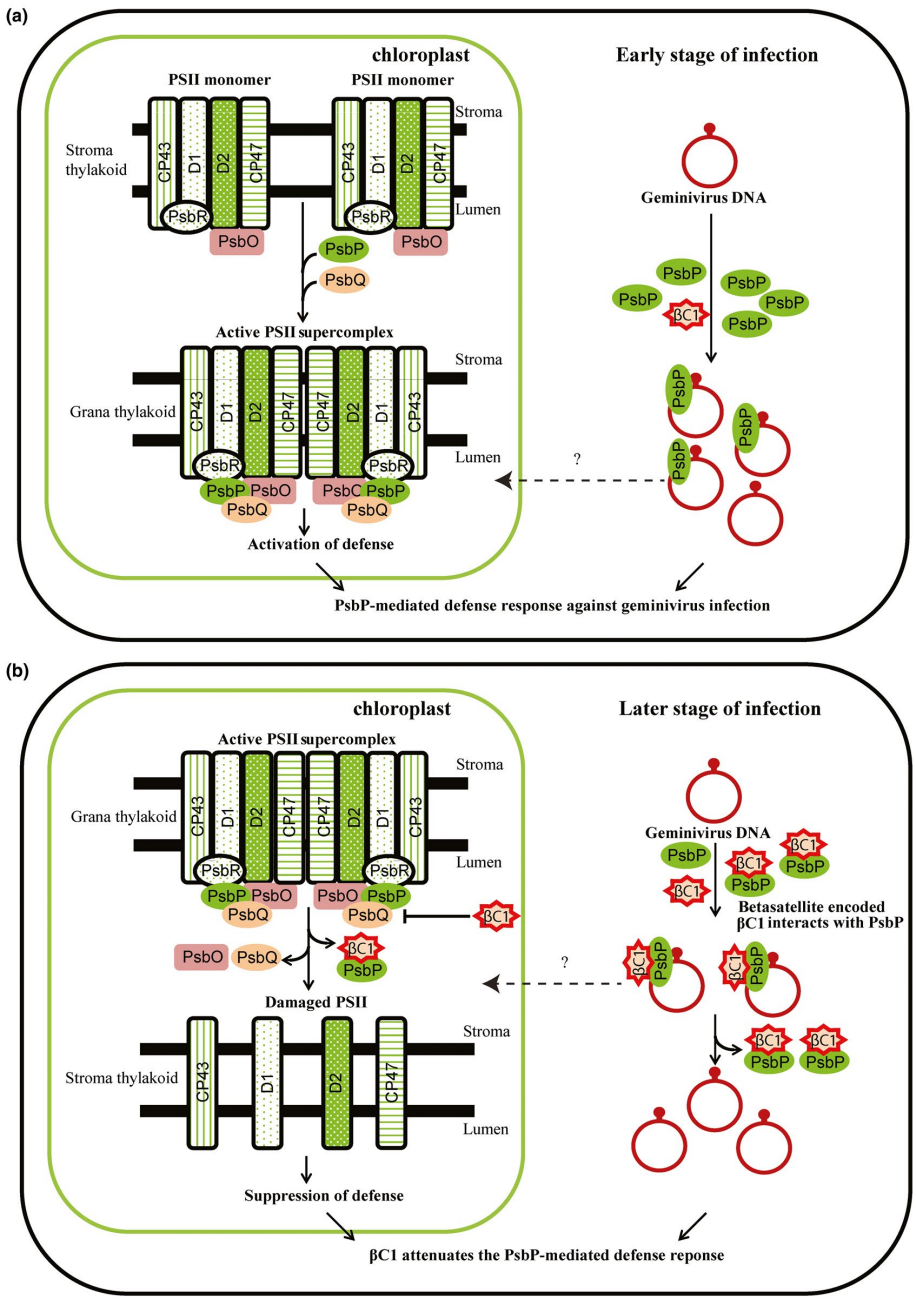
Betasatellite‐encoded βC1 protein subverts PsbP‐mediated defence response against geminivirus infection. (a) PsbP‐mediated defence response against geminivirus infection. During the early stage of viral infection, plant innate immunity necessitates the oxygen‐evolving enhancer protein 2, PsbP to exhibit an antiviral response in the infected plant cells. PsbP binding to viral DNA possibly interferes with systemic infection and/or movement of viral DNA from the infected cells. Consequently, PsbP binding to geminivirus DNA impedes symptom induction and viral DNA accumulation, thereby hampers viral pathogenesis. In addition, the OEC of PSII facilitated defence‐related reactive oxygen species (ROS) production lead to an antiviral state and subsequently impedes the viral infection cycle. (b) βC1 attenuates the PsbP‐mediated defence response during betasatellite infection. During the later stage of viral infection, βC1 protein being pathogenicity determinant targets the OEC of PSII and disturbs the photosynthetic function and ultrastructure of chloroplasts. The damage caused by βC1 on the OEC of PSII possibly suppresses defence‐related reactive oxygen species (ROS) production, subsequently exhibit compromised cell death supporting viral pathogenesis. Betasatellite‐mediated veinal chlorosis symptom induction is associated with damage in the chloroplastic structure and function. Further, βC1 protein interacts with PsbP protein and hampers its DNA binding ability. The βC1‐PsbP interaction subverts the impediment on viral pathogenesis mediated by PsbP binding to geminivirus DNA. However, precise subcellular locations of these interactions are not known.

With insights from current and previous studies, we hypothesized the working model for βC1‐PsbP interaction (Fig. 7). Our study highlighted novel DNA binding property of PsbP protein. Enhanced symptom induction and viral DNA accumulation in infected *PsbP*‐silenced *N. benthamiana* plants suggest that PsbP mediates an antiviral defence response. Furthermore, binding of PsbP protein to the geminivirus DNA in infected *N. benthamiana* plants may possibly interfere with replication, transcription and cell‐to‐cell movement of viral DNA. On the other hand, autophagy plays role in antiviral defence, in addition to its contribution to plant immunity against necrotrophic or biotrophic pathogen (Haxim *et al*., 2017). PsbP facilitates the formation and stabilization of active PSII super complex in the grana thylakoid membrane (Ido *et al*., 2009). Proper functioning of OEC and PSII leads to defence‐related ROS production against pathogen, which activates autophagy (Rodriguez‐Herva *et al*., 2012).

To overcome the defence responses generated by plants, viral encoded multifunctional proteins adopt counteracting strategies. The interaction studies showed that betasatellite‐encoded βC1 protein interacts with PsbP. PsbP overexpressing *N. benthamiana* plants showed reduced viral titre at early stage of betasatellite infection and not during the later stage. In this scenario, βC1‐PsbP interaction interferes with the DNA binding ability of PsbP and probably attenuates the PsbP‐mediated antiviral response. Although betasatellite infection damages the PSII activity, the higher rate of damage of PSII activity in transgenic *PsbP* dRNAi *N. tabacum* plants signifies the positive correlation of PsbP level and PSII activity. These results suggest that βC1‐PsbP interaction destabilizes the active PSII super complex, thereby suppressing the defence‐related ROS production and possibly exhibiting compromised cell death. The βC1‐PsbP interaction subverts the impediment on viral pathogenesis mediated by PsbP binding to geminivirus DNA.

In summary, βC1 protein interacts with host PsbP protein. Additionally, PsbP binds to both helper virus and betasatellite DNA. PsbP‐mediated antiviral response impedes symptom induction and viral DNA accumulation in geminivirus infected plants. Irrespective of PsbP isoform types, both PsbP group‐I and PsbP group‐II isoform expression negatively correlates with the betasatellite infection. However, geminivirus generated counter‐defence attenuates the PsbP‐mediated defence response during the later stage of viral infection. The βC1 protein interacts with PsbP and affects its ability of binding to viral DNA. In addition, betasatellite infection damages the ultrastructure and function of the chloroplast, thereby overcoming the plant defence response. However, PsbP‐mediated modulation of PSII activity is not solely responsible for the chloroplast ultrastructural damage caused during betasatellite infection. In conclusion, βC1 subverts the PsbP‐mediated impediment on symptom induction and viral pathogenesis during geminivirus infection. In future, the delicate regulation of host and viral protein during infection and its effect on assembly of OEC and PSII activity need to be investigated in detail.

## Experimental procedures

### Plant inoculation and source of plant material and infectious virus clones

The 35S‐PsbP transgenic *N. benthamiana* plant seeds (Kong *et al*., 2014) were kindly gifted by Professor Xueping Zhou, Institute of Biotechnology, and Zhejiang University, China. The seeds of wild‐type and stable transgenic *PsbP* dRNAi *Nicotiana tabacum* cv. Samsun NN lines, namely, IAir and 2FAir (Ishihara *et al*., 2005) were kindly provided by Dr. Kentaro Ifuku, Kyoto University, Japan. Infectious constructs of ToLCNDV (referred as A) (GenBank accession no. U15015) (Chakraborty *et al*., 2008), RaLCB (referred as β) (GenBank accession no. EF175734) (Singh *et al*., 2012), and Radish leaf curl betasatellite mutant (RaLCBHAβC1: referred as βHAβC1) (Bhattacharyya *et al*., 2015) were already available in our laboratory. Inoculation of tobacco plants (*N. benthamiana* and *N. tabacum* cv. Samsun NN) was done as previously described (Bhattacharyya *et al*., 2015). In each case, at least five plants were inoculated following standard procedures and each experiment was repeated three times. Symptom severity on test plants was recorded according to Chakraborty *et al*. (2008).

### Plasmids construction

The full‐length coding sequence of *NbPsbP1* was amplified from cDNA of *N. benthamiana* using the primer pair LOPS23FP/NBPS23RP and cloned into the pJET1.2 vector. The cloned NbPsbP1 (GenBank accession no. MK472713) showed maximum nucleotide identity of 99% with *N. benthamiana* chloroplast PsbP1 precursor mRNA (GenBank accession no. JF897607.1). To generate yeast two‐hybrid (Y2H) constructs nucleotides corresponding to the PsbP (1‐795 bp), PsbPD12 (1‐600 bp) and PsbPD1 (1‐300 bp) were Polymerase Chain Reaction (PCR) amplified from the pJET1.2‐PsbP plasmid using primer pair LOPS23FP/NBPS23RP, LOPS23FP/ PSBPD2RP and LOPS23FP/PSBPD1RP, respectively (Supplementary Table. S4) and were cloned in frame in pGADT7 vector. The βC1 open reading frame (ORF) of RaLCB was amplified with the specific primer pair LORLΒC1FP/RLΒC1RP from the RaLCB monomeric clone (Singh *et al*., 2012) and cloned into pGBKT7 vector. Similarly, the BiFC constructs were generated by amplifying PsbP (1‐795 bp) and PsbPD12 (1‐600 bp) from the pJET1.2‐PsbP clone using primer pairs PS23BIFP/NBPS23RP and PS23BIFP/PSBPD2RP, respectively and cloned into pVYCE(R) vector. Similarly, the βC1 ORF was cloned in pVYNE vector using specific primer pair RLΒC1FP/RLΒC1RP. Full‐length coding sequence of *PsbP* was amplified with PS23BIFP/PSbPEXRP primers and cloned into the pMAL‐c2X vector. The βC1 ORF of RaLCB was amplified with RLΒC1FP/RLΒC1RP primers and cloned into the pGEX‐6p‐2 vector. To generate pTRV‐*PsbP*‐silencing construct, *PsbP*‐fragment (498 bp) was amplified using the primer pair PSBPSILFP/PSbPD2RP and cloned in *Eco*RI and *Bam*HI restriction site of the pTRV2 vector. The full‐length coding sequence of *NtPsbP1* was amplified from cDNA of *N. tabacum* cv. Samsun NN using the primer pair LOPS23FP/NTPS23RP and cloned into the pGADT7 vector. The cloned NtPsbP1 (GenBank accession no. MK472714) showed maximum nucleotide identity of 97% with *N. tabacum* mRNA for photosystem II OEC 23 kDa polypeptide (GenBank accession no. X58909.1). All the primers used in this study are mentioned in Supplementary Table S4.

### Yeast two‐hybrid assay

Yeast two‐hybrid assays were carried out by co‐transforming *Saccharomyces cerevisiae* strain AH109 cells with plasmid BD‐βC1 plus AD‐PsbP/AD‐NtPsbP. AD‐T_Ag _and BD‐P_53_ act as a positive control, while AD and BD, AD‐PsbP/AD‐NtPsbP and BD, and AD and BD‐βC1 acted as negative controls. Further, the plasmids combinations BD‐βC1 plus PsbP_1‐200_, and BD‐βC1 plus PsbP_1‐100 _were co‐transformed into yeast AH109 cells. BD plus PsbP_1‐200_, and BD plus PsbP_1‐100 _served as negative control. The interaction was checked by growing the transformants on synthetic dropout media lacking Leu, Trp, His and containing 5 mM 3‐amino‐1,2,4‐triazole at 30 °C for 96 h.

### BiFC assay

BiFC assays were performed as described by Waadt *et al*. (2008). The lower epidermis of leaves of 3‐4 weeks old *N. benthamiana* plants were co‐infiltrated with *Agrobacterium tumefaciens* strain GV3101 harbouring pVYNE‐βC1 and pVYCE(R)‐PsbP constructs. After 72 h, the reconstituted Venus fluorescence was visualized under Andor Spinning Disk Confocal Microscope (Andor, Belfast, Ireland) and the Andor iQ2.7 software was used for merging and analysis of the captured fluorescence. For detection of DAPI fluorescence, 340 nM–380 nM excitation and 435 nM–485 nM emission filters were used. For Venus fluorescence, 465 nM–495 nM excitation and 520 nM–540 nM emission filters were used. Similarly, BiFC assay was carried out with pVYNE‐βC1 and pVYCE(R)‐PsbP, and pVYNE‐βC1 and pVYCE(R)‐PsbP_1‐200_ combinations.

### Recombinant protein expression, purification and GST pull‐down assay

*Escherichia coli* strain Arctic express (DE3) carrying either pGEX‐6p‐2 vector or pGEX‐6p‐2‐βC1 expression construct were used to express either GST or GST‐βC1 fusion protein, respectively. Expression of GST/GST‐βC1 fusion was achieved after 24 h of incubation at 12 °C after 0.1 mM isopropyl β‐D‐thiogalactoside (IPTG) induction. GST/GST‐βC1 proteins were further purified from the bacterial culture using glutathione resin (G‐Biosciences, St. Louis, USA). Similarly, MBP/MBP‐PsbP fusion protein was expressed and purified from *E. coli* transformed with either pMAL‐C2X vector or pMAL‐C2X‐PsbP constructs. Expression of MBP/MBP‐PsbP was optimized by incubating at 12 °C for 16 h after 0.1 mM IPTG induction. MBP and MBP‐PsbP fusion proteins were purified using Amylose resin (New England Biolabs, Massachusetts, USA) following manufacturer’s protocol.

An equal amount of bacterially purified proteins either GST and MBP or GST‐βC1 and MBP or GST and MBP‐PsbP or GST‐βC1 and MBP‐PsbP were mixed and incubated with pre‐equilibrated glutathione resin for 4 h at 4 °C. After incubation, unbound proteins were removed by washing five times with wash buffer and the bound proteins were then eluted. Then 10% of the input protein and eluted proteins were analysed by sodium dodecyl sulfate‐polyacrylamide gel electrophoresis (SDS‐PAGE) and Western blotting assay using an anti‐GST antibody (Sigma, Saint Louis, USA) and an anti‐MBP antibody (Sigma, Saint Louis, USA).

### Electrophoretic mobility shift assay

EMSAs were performed as described previously with minor modifications (George *et al*., 2014). The affinity purified MBP‐PsbP protein and GST‐βC1 was further purified by anion‐exchange chromatography using DEAE‐sepharose resin (DFF100, Sigma, Saint Louis, USA). The 24‐mer ssDNA 5′ACGCTACGCAGCAGCCTTAGCTAC3′ (referred as SCR probe), the 30‐mer ssDNA 5′GTCGACTACAGATGAACGCGTATACACATC3′ (referred as βC1 probe) and the 20‐mer ssDNA 5′ATTGGTGTCTGGAGTCCTAT3′ (referred as CR probe) were labelled with [γ‐32p] ATP using T4 polynucleotide kinase and used as radiolabelled ssDNA substrate. Purified MBP‐PsbP was incubated with reaction buffer containing 20 mM Tris‐HCl pH 8.0, 2 mM DTT, 5 mM MgCl2, 12% glycerol, 25 mM KCl, 2 mM ATP and 10 nM radiolabelled DNA‐probe for 15 min at room temperature. The reaction product was run on 3.7% native polyacrylamide gel and autoradiographed. Equal amount of purified MBP or GST protein served as negative control.

Additionally, to check the specificity of PsbP binding to betasatellite genome, either [γ‐32p] ATP radiolabelled ssDNA (corresponding to 1103 nt–50 nt of betasatellite) or [α‐32 p] dCTP radiolabelled dsDNA fragment (corresponding to 1103 bp–50 bp of betasatellite) was used as DNA‐probe. Purified MBP‐PsbP (1.0 µg) was incubated with reaction buffer containing 20 mM Tris‐HCl pH 8.0, 2 mM DTT, 5 mM MgCl_2_, 12% glycerol, 25 mM KCl, 2 mM ATP and 10 nM radiolabelled DNA‐probe for 15 min at room temperature. Also 1 µM of unlabelled 24‐mer NbActin ssDNA (5′AGGCTGGATTTGCTGGAGATGATG3′) was used as ssDNA competitor whereas 1 µM of unlabelled pMAL‐c2X DNA was used as dsDNA competitor. Protein competitive EMSA was carried using MBP‐PsbP (1.0 μg), radiolabelled 24‐mer ssDNA 5′ACGCTACGCAGCAGCCTTAGCTAC3′ (referred as SCR probe) and increasing concentration of GST‐βC1 protein (0.25 μg–1.0 μg). The competitive EMSA was performed either by addition of MBP‐PsbP protein to the EMSA reaction where the probe is pre‐incubated with increasing concentration of GST‐βC1 or *vice versa*. Purified GST protein (1.0 μg) was used a negative control for protein competitive EMSA.

### Virus‐induced gene silencing of endogenous *PsbP* of *N .benthamiana*

A *Tobacco rattle virus* (TRV)‐based gene silencing vector was used in this study for transient silencing. pTRV1 (CD3‐1039), pTRV2 (CD3‐1040) and pTRV2‐*NtPDS* (CD3‐1045) vectors (Liu *et al*., 2002) were procured from the Arabidopsis Biological Resource Center (ABRC). The pTRV1, pTRV2 and pTRV2‐*PsbP* constructs were mobilized into *A. tumefaciens* strain EHA105. Silencing of the *PsbP* gene was achieved by co‐infiltrating the lower leaves of 3 weeks old *N. benthamiana* plants with *Agrobacterium* cells carrying pTRV1 + pTRV2‐*PsbP*. Plants co‐infiltrated with *Agrobacterium* carrying pTRV1 and pTRV2 served as a negative control for *PsbP*‐silencing. Inoculated plants were grown at 22 °C, 70% relative humidity, and in 16 h of light/8 h of dark photoperiod.

### Southern and northern RNA blotting

Total DNA was extracted from the uppermost leaves of the inoculated plants and Southern blotting was performed as described previously by Singh *et al* (2012). Total RNA was extracted from the uppermost leaves of the test plants using a standard protocol (Singh *et al*., 2016). RNA blotting analysis was performed using the protocol as described earlier (Xiong *et al*., 2009). RNA samples were isolated from three different plants for each combination and the experiment was repeated three times.

### Computational analysis

BLASTp analysis with protein sequence of PsbP and βC1 against PDB (https://www.rcsb.org/) databank gave significant hits for PsbP but not for βC1. Since partial PsbP structure is available in PDB (1V2B_A) with help of in silico tools we generated the computational model. The protein structure of PsbP and βC1 is modelled using Phyre2 (http://www.sbg.bio.ic.ac.uk/phyre2/). Further, obtained 3D structures of these proteins were subjected to 20 ns MD simulation using GROMACS. DNA binding region of PsbP was predicted using the DISLPAR (DNA‐Interaction Site Prediction from a List of Adjacent Residues) tool (http://pipe.scs.fsu.edu/displar.html). Protein‐Protein interaction site of PsbP and βC1 were predicted using cons‐PPISP (consensus Protein‐Protein Interaction Site Predictor) tool (http://pipe.scs.fsu.edu/ppisp.html). The docking studies were carried out using HADDOCK server (http://milou.science.uu.nl/services/HADDOCK2.2/haddock.php).

## Authors contributions

P.G. and S.C. planned and designed the research; P.G. and K.P. performed research; P.G., K.P. and S.C analysed and interpreted data; P.G. and S.C. wrote the manuscript.

## Supporting information


**Fig. S1** Betasatellite encoded βC1 protein interacts with PsbP.

**Fig. S2** PsbP and βC1 binds to ssDNA fragment (1303‐1326) on the betasatellite genome.

**Fig. S3** βC1 protein interferes with the DNA binding activity of PsbP protein.

**Fig. S4** Predicted three dimensional (3D) structure of PsbP docked with βC1 and DNA.

**Fig. S5** TRV based PsbP silencing enhances betasatellite mediated symptom induction on *N. benthamiana* plants.

**Fig. S6** Overall expression of PsbP isoform contributes to PsbP mediated impediment against geminivirus infection.

**Fig. S7** Chloroplast ultrastructural damage caused by betasatellite is independent of PsbP mediated modulation of PSII activity.

**Table S1** Infectivity of A+β on wild type and PsbP silenced *N. benthamiana* plants.

**Table S2** Infectivity of A+β on wild type and transgenic *N. benthamiana* transgenic plants over expressing PsbP.

**Table S3** Infectivity of A+β on wild type and transgenic *PsbP* dRNAi *N. tabacum* Samsun NN plants.

**Table S4** Sequences of the primers used in this study.
