# Supplementary material for: A geminivirus betasatellite encoded βC1 protein interacts with PsbP and subverts PsbP‐mediated antiviral defence in plants
Source: Mol Plant Pathol. 2019 Apr 15;20(7):943–60. doi: 10.1111/mpp.12804 (PMC6589724; doi:10.1111/mpp.12804)
Supplement: Supplementary file 1 — Fig. S1 Betasatellite encoded βC1 protein interacts with PsbP. [file MPP-20-943-s001.doc]

**Figure S1. Betasatellite encoded βC1 protein interacts with PsbP.**

(a) The immunoprecipitation assay carried out with leaves of *N. benthamiana* plants inoculated with either A-alone or A+βHAβC1 using anti-HA antibodies. At 14 dpi, total protein was isolated from the two upper-most systemic leaves of either A-alone or A+ βHAβC1-inoculated plant. Subsequently, immunoprecipitation of HA-βC1 protein was carried out with the anti-HA antibody (Sigma, St. Louis, USA) using a standard protocol. Total protein isolated from A-inoculated plants was used as negative control. The 23kDa protein was found to specifically immunoprecipitated with HA-βC1. The sequencing result of 23kDa protein band by MALDI-TOF analysis matches with chloroplast photosynthetic oxygen evolving protein 23kDa subunit. (b) Yeast cells were cotransformed with different combinations of yeast two-hybrid constructs as indicated. Protein-protein interactions were tested by ability of the cotransformed to grow on selection medium SD-Leu-Trp-His supplemented with 5mM 3-amino-1,2,4-triazole. Yeast cells were plated as serial dilution from cultures OD600 of 1.0. Yeast cells cotransformed with AD plus BD, and AD-TAg plus BDP53 were used as negative and positive controls, respectively.

**
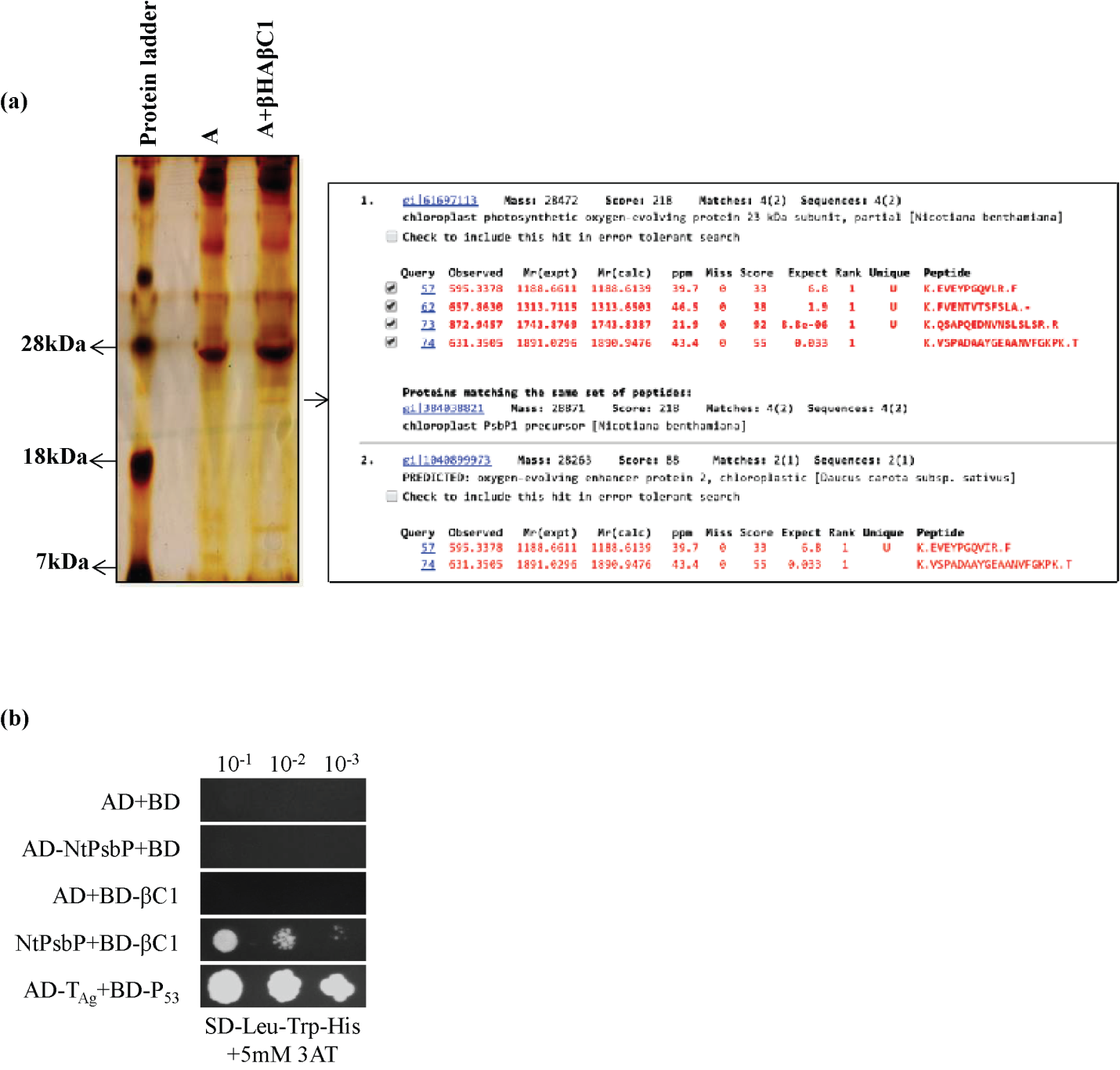
**
