# Supplementary material for: A geminivirus betasatellite encoded βC1 protein interacts with PsbP and subverts PsbP‐mediated antiviral defence in plants
Source: Mol Plant Pathol. 2019 Apr 15;20(7):943–60. doi: 10.1111/mpp.12804 (PMC6589724; doi:10.1111/mpp.12804)
Supplement: Supplementary file 2 — Fig. S2 PsbP and βC1 binds to ssDNA fragment (1303‐1326) on the betasatellite genome. [file MPP-20-943-s002.doc]

**Figure S2. PsbP and βC1 binds to ssDNA fragment located in the betasatellite genome.**

The autoradiograph of electrophoretic mobility shift assay (EMSA) showing binding of MBP-PsbP (a) and GST-βC1 (b) with SCR probe (1303-1326nt). Either purified MBP or GST protein was used as negative control.

**
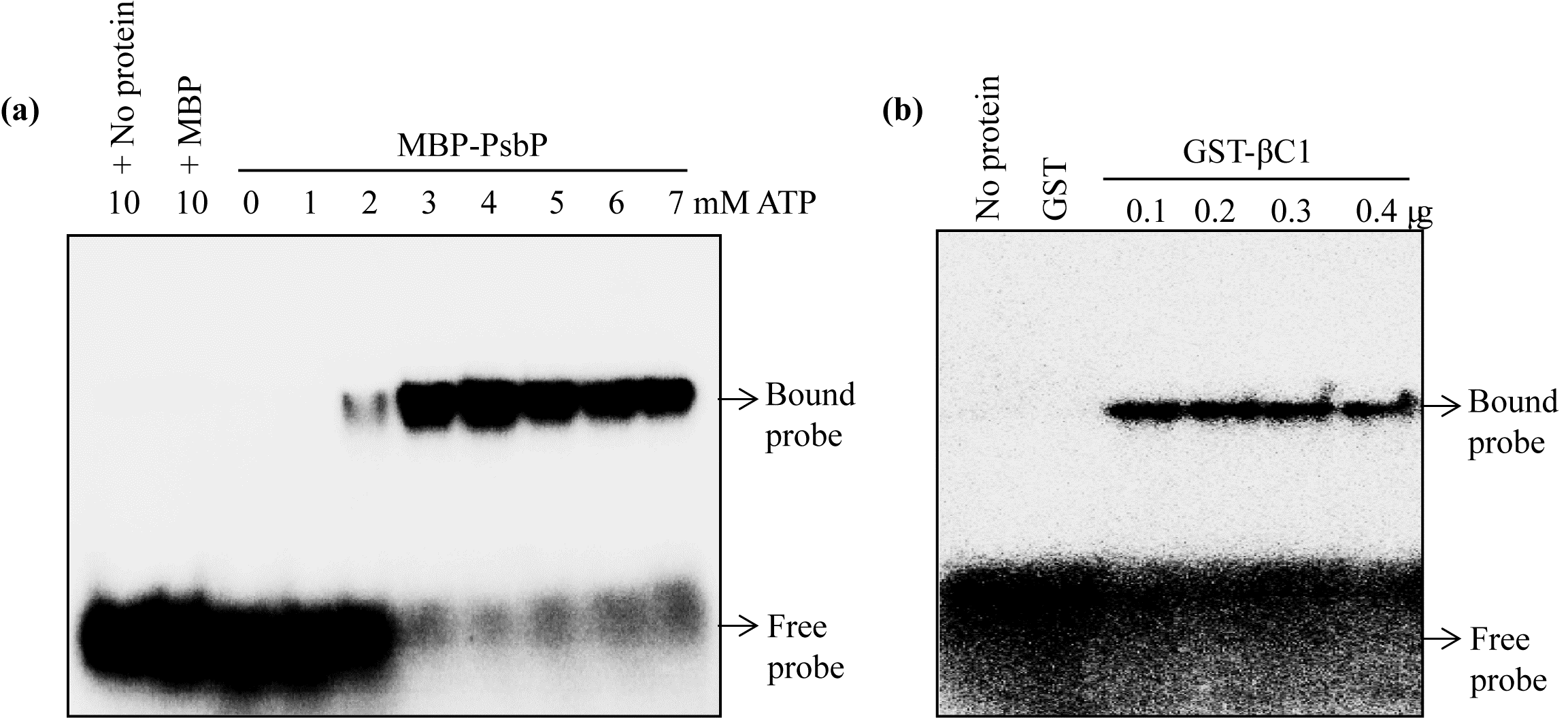
**
