# Supplementary material for: A geminivirus betasatellite encoded βC1 protein interacts with PsbP and subverts PsbP‐mediated antiviral defence in plants
Source: Mol Plant Pathol. 2019 Apr 15;20(7):943–60. doi: 10.1111/mpp.12804 (PMC6589724; doi:10.1111/mpp.12804)
Supplement: Supplementary file 3 — Fig. S3 βC1 protein interferes with the DNA binding activity of PsbP protein. [file MPP-20-943-s003.doc]

**Figure S3. βC1 protein interferes with the DNA binding activity of PsbP protein**.

(a) Autoradiograph of protein competitive EMSA performed by incubating MBP-PsbP protein with DNA probe (SCR-region 1303-1326nt) pre-incubated with increasing concentration ofGST-βC1 protein. (b) Autoradiograph of protein competitive EMSA performed by incubating increasing concentration of GST-βC1 protein to DNA probe (SCR-region, 1303-1326nt) pre-incubated with MBP-PsbP protein. Purified GST protein was used as negative control in thecompetitive EMSA.

**
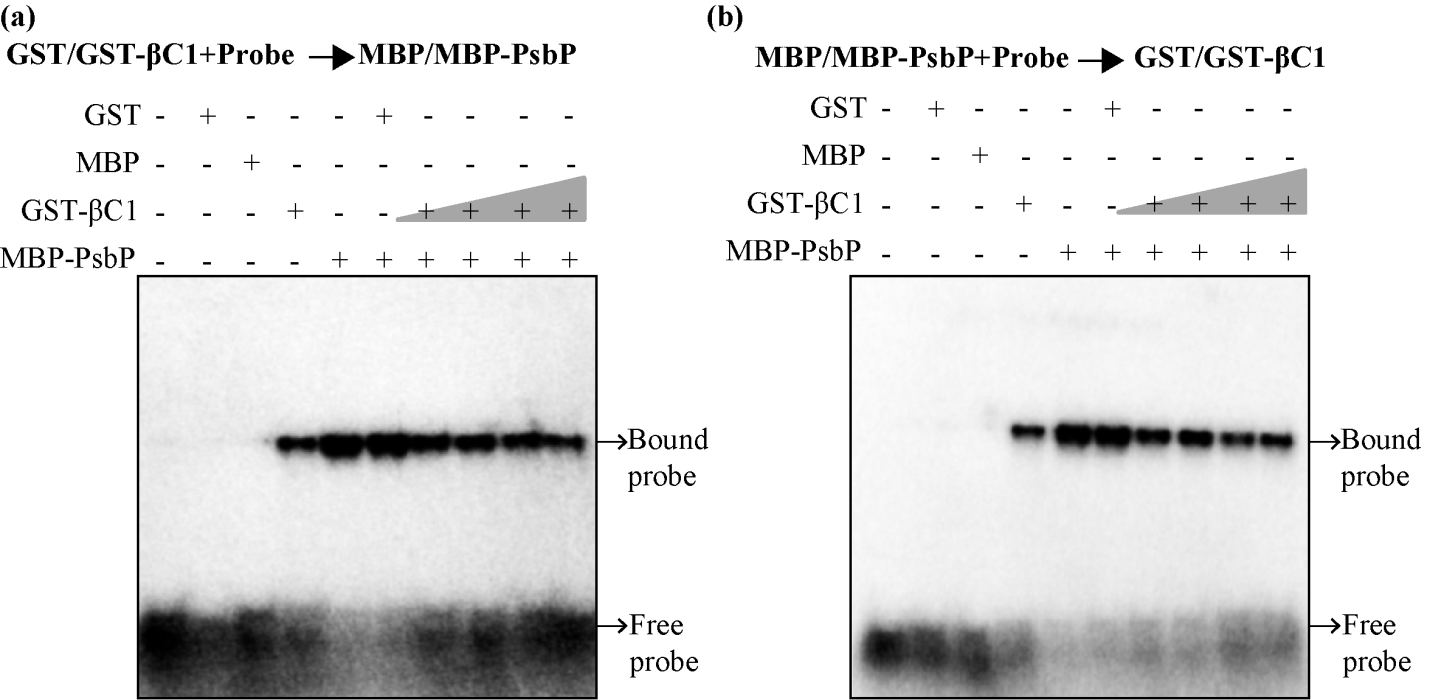
**
