# Supplementary material for: A geminivirus betasatellite encoded βC1 protein interacts with PsbP and subverts PsbP‐mediated antiviral defence in plants
Source: Mol Plant Pathol. 2019 Apr 15;20(7):943–60. doi: 10.1111/mpp.12804 (PMC6589724; doi:10.1111/mpp.12804)
Supplement: Supplementary file 4 — Fig. S4 Predicted three dimensional (3D) structure of PsbP docked with βC1 and DNA. [file MPP-20-943-s004.doc]

**Figure S4. Predicted three-dimensional (3D) structure of PsbP docked with βC1 and DNA.**

The interacting residues of the PsbP with βC1 (a), ssDNA (b) and dsDNA (c) are presented as enlarged view.(d) In silico docking of dsDNA with PsbP and PsbP-βC1 complex. (e) In silico docking to identify the residues of PsbP that interacts with either βC1 protein or dsDNA.

**
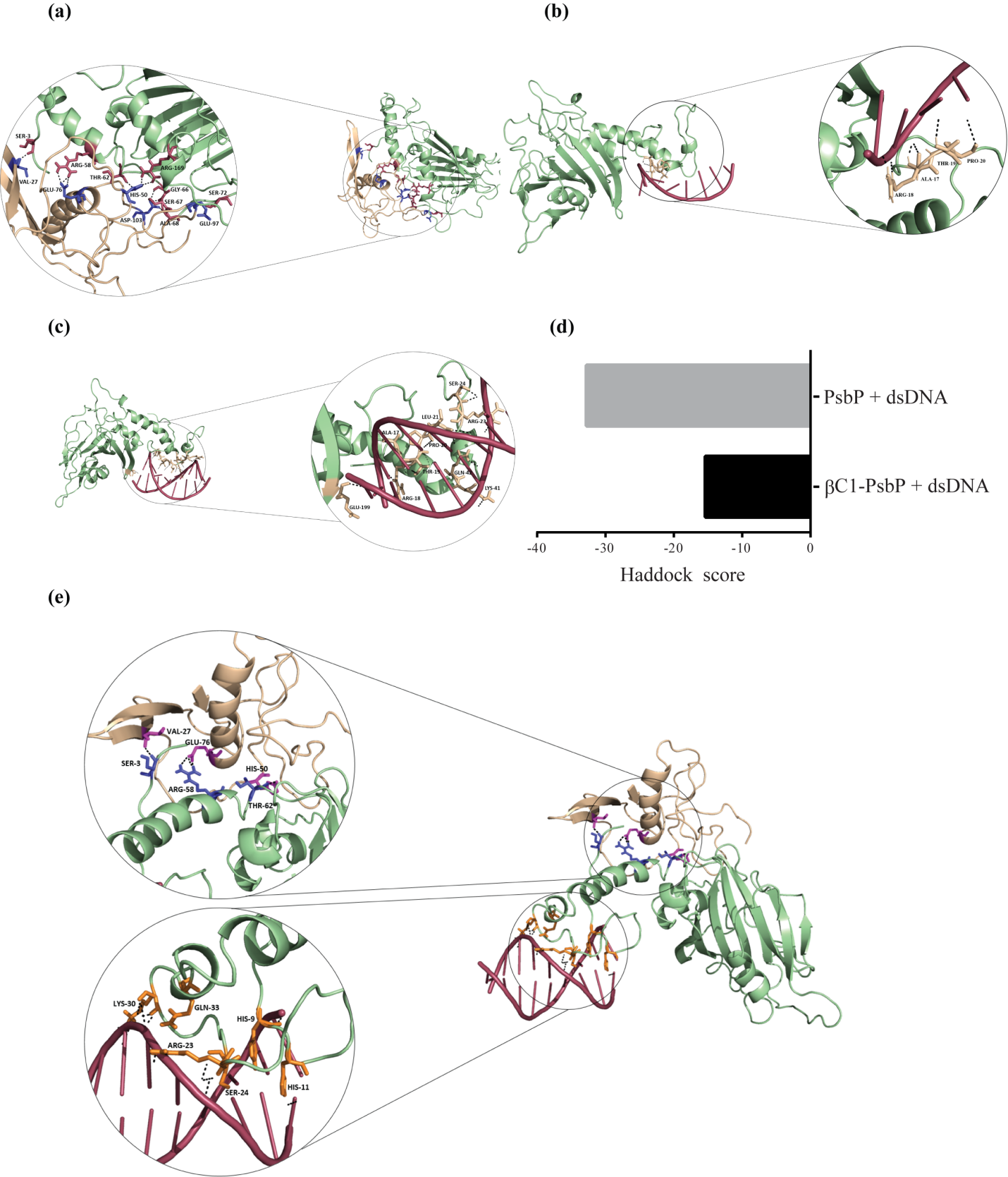
**
