# Supplementary figures and images for: A geminivirus betasatellite encoded βC1 protein interacts with PsbP and subverts PsbP‐mediated antiviral defence in plants
Source: Mol Plant Pathol. 2019 Apr 15;20(7):943–60. doi: 10.1111/mpp.12804 (PMC6589724; doi:10.1111/mpp.12804)

**Figure S5**

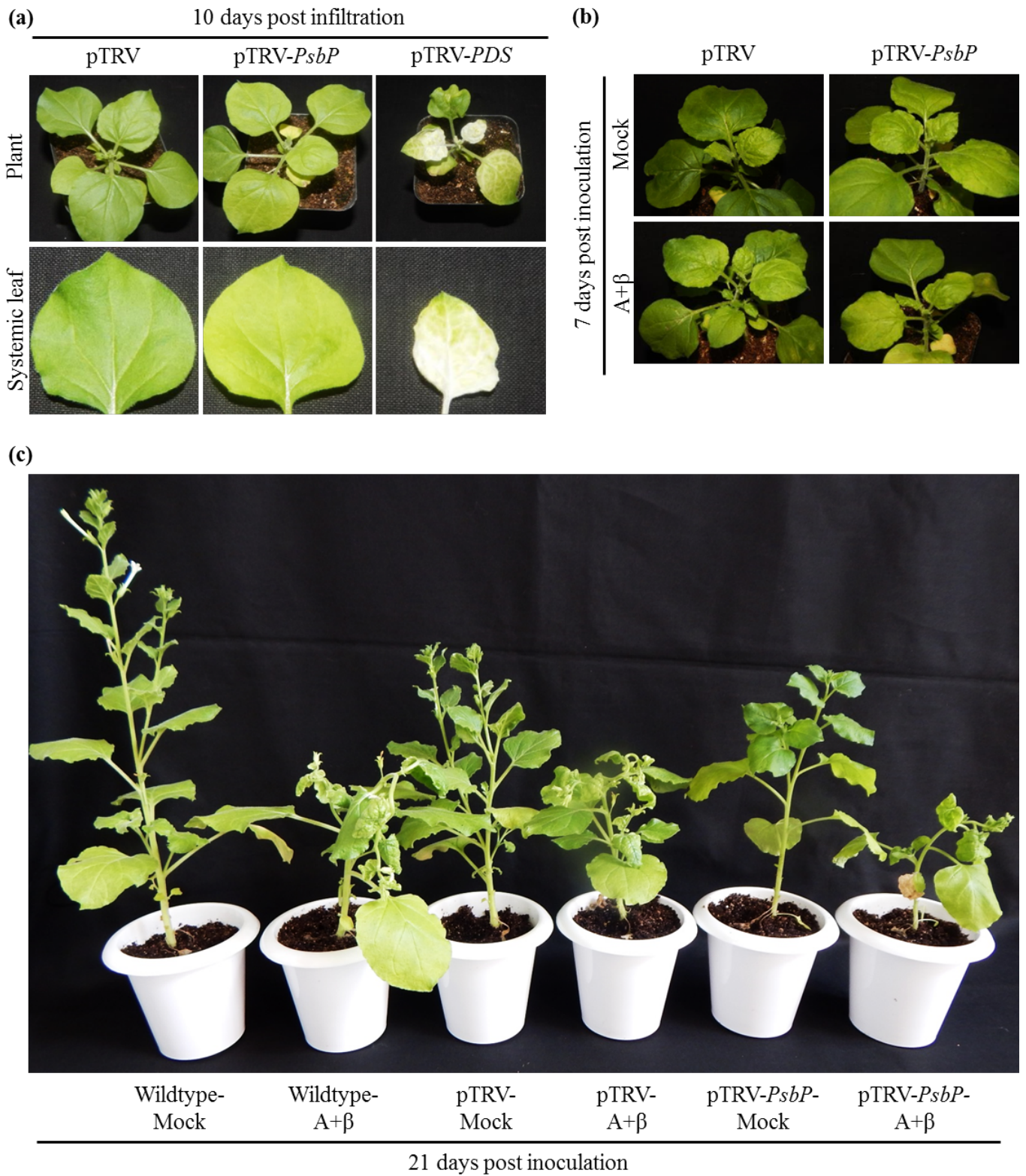

Supplement: Supplementary file 5 — Fig. S5 TRV based PsbP silencing enhances betasatellite mediated symptom induction on N. benthamiana plants. [file MPP-20-943-s005.pdf]
