# Supplementary material for: A geminivirus betasatellite encoded βC1 protein interacts with PsbP and subverts PsbP‐mediated antiviral defence in plants
Source: Mol Plant Pathol. 2019 Apr 15;20(7):943–60. doi: 10.1111/mpp.12804 (PMC6589724; doi:10.1111/mpp.12804)
Supplement: Supplementary file 6 — Fig. S6 Overall expression of PsbP isoform contributes to PsbP mediated impediment against geminivirus infection. [file MPP-20-943-s006.doc]

**Figure S6. Overall expression of PsbP isoform contributes to PsbP-mediated impediment against geminivirus infection.**

Southern blotting analysis shows comparative level of DNA A at 14dpi (a) and 21dpi (b) in wild-type, 1Air, and 2FAir *N. tabacum* plants.

**
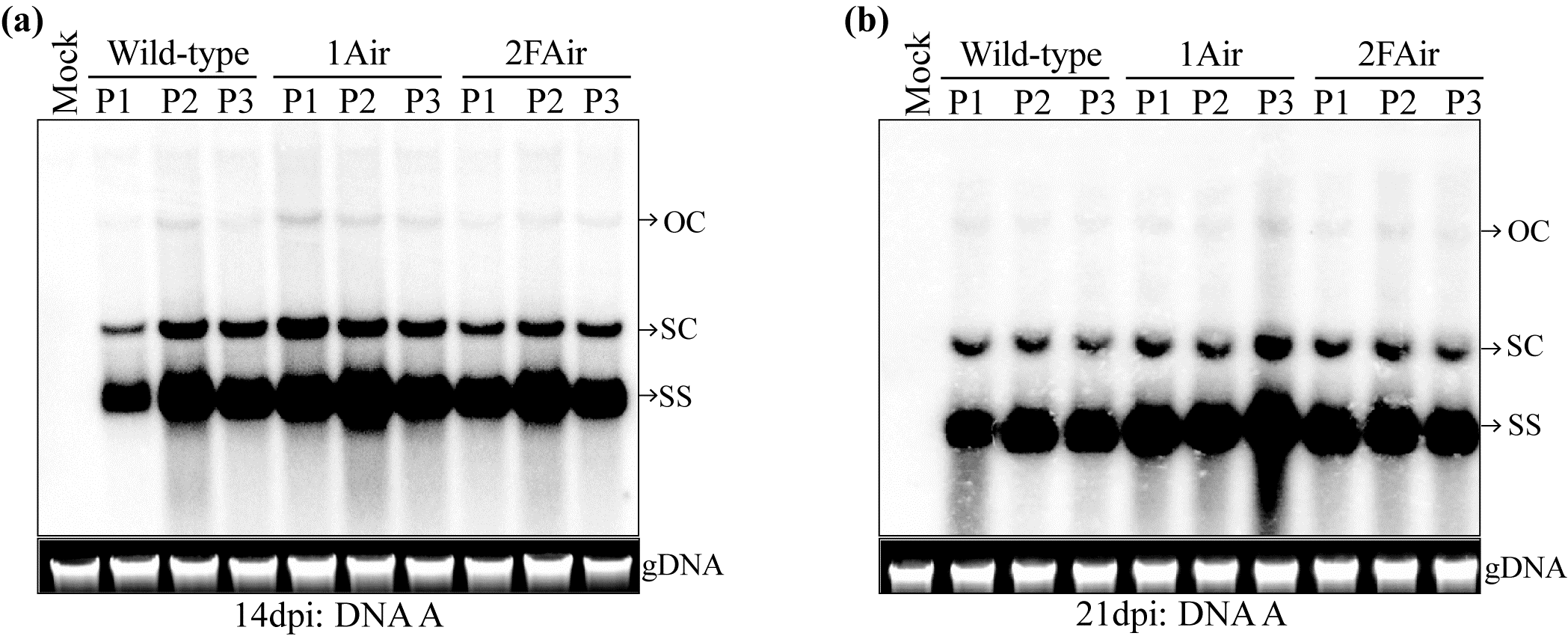
**
