# Supplementary material for: A geminivirus betasatellite encoded βC1 protein interacts with PsbP and subverts PsbP‐mediated antiviral defence in plants
Source: Mol Plant Pathol. 2019 Apr 15;20(7):943–60. doi: 10.1111/mpp.12804 (PMC6589724; doi:10.1111/mpp.12804)

**Figure S7. Chloroplast ultrastructural damage caused by betasatellite is independent of PsbP-mediated modulation of PSII activity.**

(a) Maximum quantum efficiency of PSII was calculated from either mock- or A+β inoculated wild-type, 1Air, and 2FAir *N. tabacum* plants at 14dpi. Asterisks indicate samples that are statistically significant in comparison with A+β wild-type plants. (*, P<0.05; **, P<0.01; ***, P<0.001) as determined by Dunnett's multiple comparisons test by ANOVA. Values are mean± SD of three independent biological and technical replicates. (b) Chloroplast ultrastructure and thylakoid organization of either mock- or A+β inoculated wild-type and transgenic 1Airand 2FAir *N. tabacum* plants as observed by transmission electron microscopy. An arrow (→) indicates disappearance thylakoid membrane and damaged grana stacking; an open circle (O) indicates a swollen thylakoid. Scale bar represents 100nm.


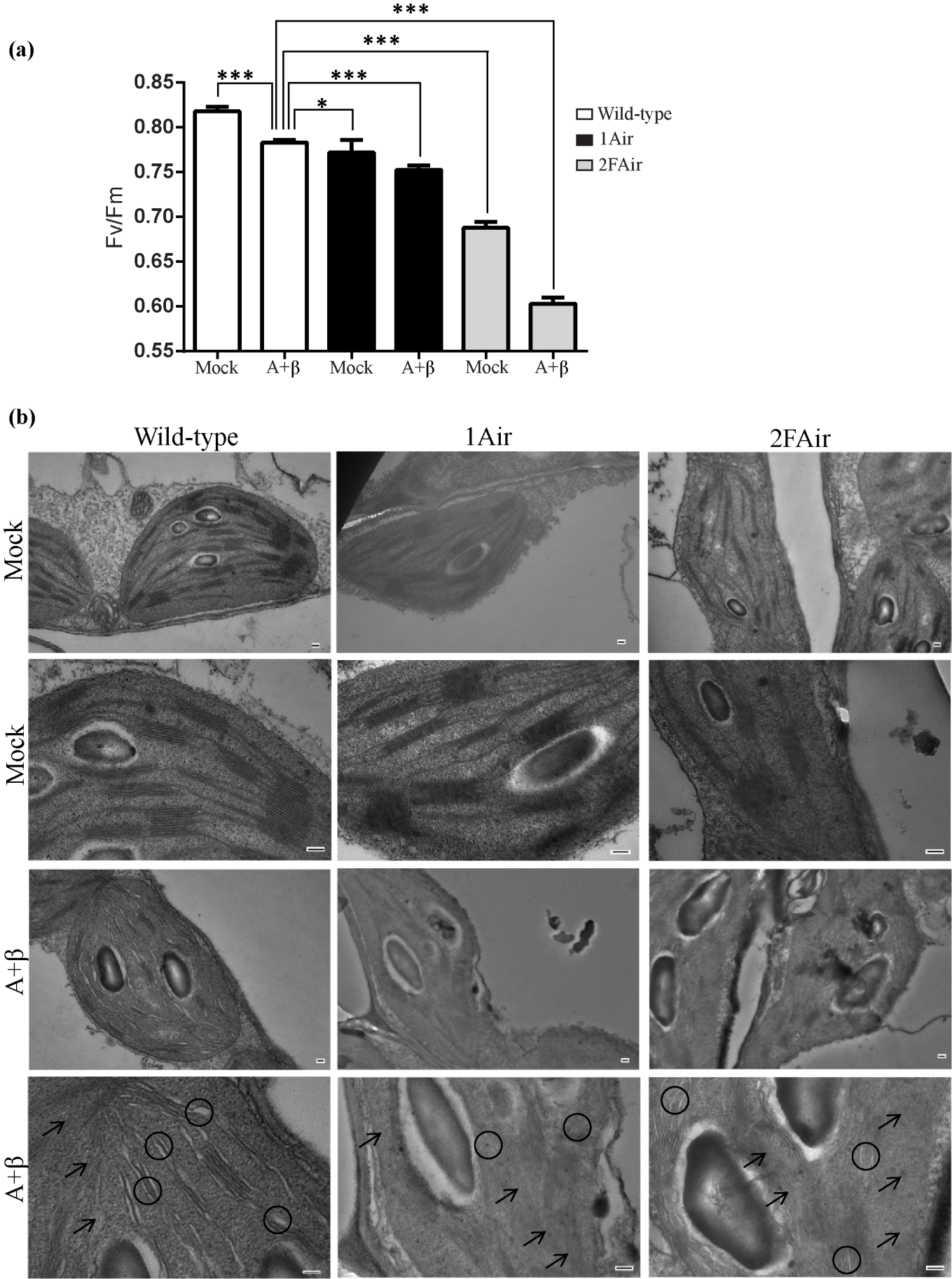

Supplement: Supplementary file 7 — Fig. S7 Chloroplast ultrastructural damage caused by betasatellite is independent of PsbP mediated modulation of PSII activity. [file MPP-20-943-s007.doc]
