# Supplementary material for: A geminivirus betasatellite encoded βC1 protein interacts with PsbP and subverts PsbP‐mediated antiviral defence in plants
Source: Mol Plant Pathol. 2019 Apr 15;20(7):943–60. doi: 10.1111/mpp.12804 (PMC6589724; doi:10.1111/mpp.12804)
Supplement: Supplementary file 8 — Table S1 Infectivity of A+β on wild type and PsbP silenced N. benthamiana plants. [file MPP-20-943-s008.doc]

**Table S1. Infectivity of A+β on wild-type and *PsbP-*silenced *N. benthamiana* plants.**

| **Test plants** | **No of inoculated plants** | **No of symptomatic plants** | **Days to first symptom appearance** | *** Symptoms** |
| --- | --- | --- | --- | --- |
| Wild-type - Mock | 18 | 0 | - | No |
| Wild-type - A+β | 18 | 18 | 13 | LC, VT, VC, St, SB |
| TRV - Mock | 18 | 0 | - | No |
| TRV - A+β | 18 | 18 | 13 | LC, VT, VC, St, SB |
| TRV: PsbP - Mock | 18 | 0 | - | No |
| TRV: PsbP - A+β | 18 | 18 | 8 | LC, VT, VC, St, SB |

*LC - leaf curling, VT - vein thickening, VC – vein clearing, St – stunting, SB - stem bending. Initially 20 days old *N. benthamiana* plants were infiltrated for transient silencing of *NbPsbP* gene. Subsequently, either wild-type, TRV-infiltrated or NbPsbP-silenced plants (30 days old) were inoculated with either pCAMBIA2300 (as mock) or A+β.
