# Supplementary material for: A geminivirus betasatellite encoded βC1 protein interacts with PsbP and subverts PsbP‐mediated antiviral defence in plants
Source: Mol Plant Pathol. 2019 Apr 15;20(7):943–60. doi: 10.1111/mpp.12804 (PMC6589724; doi:10.1111/mpp.12804)
Supplement: Supplementary file 9 — Table S2 Infectivity of A+β on wild type and transgenic N. benthamiana transgenic plants over expressing PsbP. [file MPP-20-943-s009.doc]

**Table S2. Infectivity of A+β on wild-type and transgenic *N. benthamiana plants* over-expressing PsbP.**

| **Test plants** | **No of inoculated plants** | **No of symptomatic plants** | **Days to first symptom appearance** | *** Symptoms** |
| --- | --- | --- | --- | --- |
| Wild-type - Mock | 18 | 0 | - | No |
| Wild-type - A+β | 18 | 18 | 9 | LC, VT, VC, St, SB |
| 35S: PsbP - Mock | 18 | 0 | - | No |
| 35S: PsbP - A+β | 18 | 18 | 11 | LC, VT, VC, St, SB |

*LC - leaf curling, VT - vein thickening, VC – vein clearing, St - stunting, SB - stem bending. Fourteen days old *N. benthamiana* plants (3-4 leaf stage) were used for either mock or A+β inoculation.
