# Supplementary material for: A geminivirus betasatellite encoded βC1 protein interacts with PsbP and subverts PsbP‐mediated antiviral defence in plants
Source: Mol Plant Pathol. 2019 Apr 15;20(7):943–60. doi: 10.1111/mpp.12804 (PMC6589724; doi:10.1111/mpp.12804)
Supplement: Supplementary file 10 — Table S3 Infectivity of A+β on wild type and transgenic PsbP dRNAi N. tabacum Samsun NN plants. [file MPP-20-943-s010.doc]

**Table S3. Infectivity of A+β on wild-type and transgenic *PsbP* dRNAi *N. tabacum* Samsun NN plants**

| **Test plants** | **No of inoculated plants** | **No of symptomatic plants** | **Days to first symptom appearance** | *** Symptoms** |
| --- | --- | --- | --- | --- |
| Wild-type - Mock | 15 | 0 | - | No |
| Wild-type - A+β | 36 | 22 | 12 | LC, VT, VC, SB |
| 1Air - Mock | 15 | 0 | - | No |
| 1Air - A+β | 36 | 12 | 9 | LC, VT, VC, SB |
| 2FAir - Mock | 15 | 0 | - | No |
| 2FAir - A+β | 36 | 10 | 10 | LC, VT, VC, SB |

*LC - leaf curling, VT - vein thickening, VC – vein clearing, SB - stem bending. Fourteen days old tobacco plants were used for either mock or A+β inoculation.
