# Supplementary material for: A geminivirus betasatellite encoded βC1 protein interacts with PsbP and subverts PsbP‐mediated antiviral defence in plants
Source: Mol Plant Pathol. 2019 Apr 15;20(7):943–60. doi: 10.1111/mpp.12804 (PMC6589724; doi:10.1111/mpp.12804)
Supplement: Supplementary file 11 — Table S4 Sequences of the primers used in this study. [file MPP-20-943-s011.doc]

Table S4. Sequences of the primers used in this study

| **S.NO** | **Primer name** | **Primer sequence** |
| --- | --- | --- |
| 1. | LOPS23FP | 5ˈ GGATCCCAATGGCTTCCACACAATGCTTC 3ˈ |
| 2. | NBPS23RP | 5ˈ CTCGAGAGCAAGGCTGAAAGAAGTGAC 3ˈ |
| 3. | PSBPD1RP | 5ˈ CTCGAGGTATGTCTGGAAATCTGTGTC 3ˈ |
| 4. | PSBPD2RP | 5ˈ CTCGAGGACTTCTGCACTAGATGTCTCC 3ˈ |
| 5. | PS23BIFP | 5ˈ GGATCCATGGCTTCCACACAATGCTTC 3ˈ |
| 6. | NTPSBPRP | 5ˈ CTCGAGTTAAGCAAGACTGAAAGAAGTG 3ˈ |
| 7. | PSBPEXRP | 5ˈ GTCGACTTAAGCAAGGCTGAAAGAAGTGACAG 3ˈ |
| 8. | PSBPSILFP | 5ˈ TGCCGTGCCCAAAAACAGTCTGC 3ˈ |
| 9. | LORLΒC1FP | 5ˈ GGATCCCAATGACGATCAAATACAAAAACC 3ˈ |
| 10. | RLΒC1FP | 5ˈ GGATCCATGACGATCAAATACAAAAACC 3ˈ |
| 11. | RLΒC1RP | 5ˈ GTCGACTACAGATGAACGCGTATACACATC 3ˈ |
| 12. | ssDNA | 5ˈCTAAAACTCGTCGGAACTCCGATTAAGGCACTTCCGGTCACCAATTTGCGACACGCGCGGCGGTGCGTACCCCTGGGAGGGTAGGGTACCACTACGCTACGCAGCAGCCTTAGCTA 3ˈ |
